# Radiosensitization and a Less Aggressive Phenotype of Human Malignant Glioma Cells Expressing Isocitrate Dehydrogenase 1 (IDH1) Mutant Protein: Dissecting the Mechanisms

**DOI:** 10.3390/cancers11060889

**Published:** 2019-06-25

**Authors:** Jacqueline Kessler, Tim Hohmann, Antje Güttler, Marina Petrenko, Christian Ostheimer, Urszula Hohmann, Matthias Bache, Faramarz Dehghani, Dirk Vordermark

**Affiliations:** 1 Department of Radiotherapy, Faculty of Medicine, Martin Luther University Halle-Wittenberg, Ernst-Grube-Str. 40, 06097 Halle (Saale), Germany; antje.guettler@uk-halle.de (A.G.); marina.petrenko@uk-halle.de (M.P.); christian.ostheimer@uk-halle.de (C.O.); matthias.bache@uk-halle.de (M.B.); dirk.vordermark@uk-halle.de (D.V.); 2 Department of Anatomy and Cell Biology, Faculty of Medicine, Martin Luther University Halle-Wittenberg, Große Steinstraße 52, 06108 Halle (Saale), Germany; tim.hohmann@medizin.uni-halle.de (T.H.); urszula.grabiec@medizin.uni-halle.de (U.H.); faramarz.dehghani@medizin.uni-halle.de (F.D.)

**Keywords:** isocitrate dehydrogenase 1, IDH1, IDH1^R132H^, glioma, glioblastoma, cell stiffness, atomic force microscopy

## Abstract

The presence of an isocitrate dehydrogenase 1 (IDH1) mutation is associated with a less aggressive phenotype, increased sensitivity to radiation, and increased overall survival in patients with diffuse glioma. Based on in vitro experimentations in malignant glioma cell lines, the consequences on cellular processes of IDH1^R132H^ expression were analyzed. The results revealed that IDH1^R132H^ expression enhanced the radiation induced accumulation of residual γH2AX foci and decreased the amount of glutathione (GSH) independent of the oxygen status. In addition, expression of the mutant IDH1 caused a significant increase of cell stiffness and induced an altered organization of the cytoskeleton, which has been shown to reinforce cell stiffness. Furthermore, IDH1^R132H^ expression decreased the expression of vimentin, an important component of the cytoskeleton and regulator of the cell stiffness. The results emphasize the important role of mutant IDH1 in treatment of patients with diffuse gliomas especially in response to radiation. Hence, detection of the genetic status of IDH1 before therapy massively expands the utility of immunohistochemistry to accurately distinguish patients with a less aggressive and radiosensitive IDH1-mutant diffuse glioma suitable for radiotherapy from those with a more aggressive IDH1-wildtype diffuse glioma who might benefit from an individually intensified therapy comprising radiotherapy and alternative medical treatments.

## 1. Introduction

Gliomas, primary tumors of the central nervous system (CNS) are relatively rare and form a heterogeneous group of neoplasms [1]. For multiple decades the histology of gliomas was the gold standard for classification and for assessment of prognosis or therapeutic management. Molecular characterization was mainly provided as supplementary information within these histologically defined categories [2]. Due to the increasing knowledge of molecular alterations in tumors of the CNS the revised fourth edition of the World Health Organization (WHO) Classification of CNS tumors (published in 2016) included molecular surrogates, which greatly expanded the utility of immunohistochemistry for providing diagnostic, prognostic, and predictive aid in the workup of gliomas [2,3,4,5]. Nowadays, classification of gliomas encompasses two principle subgroups: diffuse gliomas, displaying an extensive infiltration in the CNS parenchyma and the nondiffuse gliomas, showing a more circumscribed growth pattern.

Immunohistochemically gliomas are categorized according to their histologically equivalent normal cell type. Diffuse gliomas are historically allocated to diffuse astrocytomas (with glioblastoma as its most frequent and most malignant representative), oligodendrogliomas, or to tumors with a mixed astrocytic and oligodendroglial phenotype (oligoastrocytomas). The group of nondiffuse gliomas now includes pilocytic astrocytoma, subependymal giant cell astrocytoma (SEGA), pleomorphic xanthoastrocytoma (PXA), and anaplastic PXA as distinct entities [2,3,4,5]. Diffuse gliomas are the most common subtype of primary brain tumors, especially in adult patients [4,6]. Within the subgroups, based on the presence/absence of marked mitotic activity, necrosis and florid microvascular proliferation diffuse gliomas are graded as WHO grade II (low-grade), III (anaplastic), or IV (glioblastoma) [4,6]. Thereby, glioblastoma can occur as the result of progression from lower grade diffuse gliomas or can arise de novo. Histologically, both primary (de novo) and secondary glioblastomas seem identical [7].

Over the last decade the understanding of glioma tumorigenesis was substantially increased due to the discovery of mutations involving the genes encoding isocitrate dehydrogenase 1 and 2 (IDH1/IDH2) enzymes. Using whole-genome sequencing and mutational analysis, Parson and colleagues identified recurrent mutations in IDH1 and IDH2 at high frequencies in WHO grade II and III astrocytomas, oligodendrogliomas, oligoastrocytomas, as well as in glioblastomas [8,9]. In these cases IDH mutations seem to predispose a particular path for oncogenic progression resulting in an increased progression-free and overall survival of affected patients, irrespective of tumor malignancy [8,10,11]. In several studies the IDH1 mutation has proven to be a powerful prognostic factor in diffuse gliomas, irrespective of tumor grade and histology [12,13,14]. Mutations in the IDH genes are generally heterozygous missense substitutions, which remarkably occur in a mutually-exclusive manner affecting only the active sites of the enzymes [8,15]. IDH1 mutations always appear in the arginine residue at codon 132 resulting in a substitution of histidine for arginine (R132H) in over 90% of all IDH1 mutations [8,16]. 

Very early on, IDH1 mutations have successfully been linked to prognostic information. Besides, it was evident that the clinical outcome for tumors with identical histology was different for IDH1-wildtype and IDH1-mutant diffuse gliomas [2,8,9,15,17,18]. Furthermore, it has been shown that many histologically identified as WHO grade II and especially WHO grade III IDH1-wildtype diffuse gliomas in adults display molecular characteristics and behaviors of a glioblastoma [13,19,20,21]. These fundamental observations drive toward a molecular classification and have led to the decision to include IDH mutation as a crucial marker for the classification of diffuse gliomas. Importantly, in this histological–molecular classification the genetic characteristics of IDH-mutant glioma can actually override the histological diagnosis, which also leads to an reclassification of glioblastoma [2]. Based on the genetic status of IDH1, glioblastomas are now divided into IDH1-wildtype glioblastoma and IDH-mutant glioblastoma, whereas the latter one largely overlaps with secondary glioblastoma in older classifications [2].

Despite the central role of an IDH1 mutation in the current classification of diffuse gliomas, it appears reasonable to further focus on cellular functions and therapeutic effects that are influenced by an IDH1 mutation. The care of patients with diffuse gliomas is challenging. Especially high-grade gliomas (grade III/IV), with glioblastoma as its most frequent and most malignant representative, are aggressive, invasive, and exhibit intratumoral hypoxia [22,23,24]. Irrespective of the multimodal therapy options available, comprising surgery, radiotherapy and chemotherapy, high-grade gliomas remain lethal diseases with dismal prognosis [25,26]. Due to their intratumoral heterogeneity and various mutual signatures (e.g., IDH mutation/1p19q co-deletion status, MGMT promoter methylation status, TERT promoter mutations) the chances for establishing a universal standard treatment of diffuse gliomas are limited [27]. In this context, the identification, but also dissection of the mechanism of molecular markers represents a useful tool for understanding cancer biology and based on this for the development of tailored therapeutic options. Furthermore, such biomarkers permit a subclassification of diffuse gliomas, making it possible to differentiate between patients with higher risk for toxicity and those who may benefit from a particular treatment i.e. in some cases, molecular markers are able to guide treatment decisions [28].

Hence, various studies highlighted the physiological roles of IDH enzymes as well as the biochemical and cellular consequences of an altered genetic status of IDH genes. IDH enzymes catalyze the decarboxylation of isocitrate to α-ketoglutarate (α-KG). Thereby, IDH1 is involved in a variety of cellular processes, including glutamine metabolism, glucose sensing and lipid metabolism, synthesis of N-acetylated amino acids, and regulation of the cellular redox status via GSH [29,30,31]. Mutations in the active sites of the IDH1 enzyme cause a distinctly decreased enzyme activity to isocitrate and result in a neomorphic enzyme function, which catalyzes the NADPH (nicotinamide adenine dinucleotide phosphate hydrogen)-dependent reduction of α-KG to the 2-hydroxyglutarate (2-HG) enantiomer, D-2-hydroxyglutarate (D-2-HG). In turn, this leads to D-2-HG accumulation and lowering α-KG as well as NADPH levels [32,33,34,35]. However, NADPH is necessary for the regeneration of reduced GSH which functions as the main antioxidant in mammalian cells. Low levels of cytoplasmic NADPH have been linked to elevated oxidative stress through impaired reduction of GSH [36]. In general, oxidative stress is increased by irradiation and chemotherapy leading to the hypothesis that IDH1 mutations induce an enhanced response to therapy and may contribute to the prolonged survival of patients harboring the mutation [37]. Due to the fact that almost all patients with a malignant glioma receive a single treatment or a combined therapy it is difficult to specify whether IDH1 mutation is associated with a less aggressive phenotype or directly linked to increased sensitivity to therapy. Thus, different studies or clinical trials have been focused on the effect of expression of mutated IDH1 on cellular behavior and response to therapy since the first mutations of IDH1 were discovered in 2008 [17,38,39,40]. 

In previous studies and by using transduced malignant glioma cell lines U-251MG (glioblastoma) U-343MG (anaplastic astrocytoma) and LN-229 (glioblastoma) we showed that gene expression of mutated IDH1 (IDH1^R132H^) resulted in elevated radiosensitivity [41,42,43]. Furthermore, gene expression of IDH1^R132H^ caused a reduced aggressiveness based on slightly decreased cell proliferation and plating efficiency, altered growth properties in 3D spheroid culture and significantly reduced cell migration in these glioma cell lines [41]. In addition, the effect of gene expression of mutated IDH1 on the radiosensitivity and cellular behavior was independent of the oxygen concentrations [41].

Based on the complex role of an IDH1 mutation in progression, aggressive biological behavior and response to therapy of malignant diffuse gliomas, it appears reasonable to dissect the molecular mechanisms underlying the less aggressive phenotype and increased sensitivity to radiation of IDH1R132H-gliomas.

## 2. Results

### 2.1. Expression of IDH1^R132H^ Enhanced the Radiation Induced Accumulation of Residual γH2AX Foci 

In eukaryotic cells, DNA double-strand breaks (DSBs) occur frequently from endogenous cellular processes or are caused by exogenous sources such as ionizing radiation. In response to the introduction of DNA DSBs, the minor histone H2A variant is rapidly phosphorylated on Ser-139 to produce γH2AX [44,45]. Based on the direct correlation between the number of DSBs and γH2AX foci, quantitation of γH2AX foci formation was applied as a marker of DNA damage and repair [44,46]. Foci which persist for longer than 24 h, so-called residual γH2AX foci, indicate unrepaired or misrepaired DSBs. These unsuccessfully repaired DSBs are generally assumed to play a major role in radiation‑induced cell death [47]. Previous studies have demonstrated a linear relationship between radiation dose and the number of γ-H2AX foci over a limited dose range (between 0.001 and 2 Gy) [48,49,50,51]. Therefore, analyzing the induction of γH2AX foci allows for the indirect evaluation an influence of IDH1^R132H^ gene expression on the effect of radiation in U-251MG, U-343MG, and LN-229 cells.

For this purpose, untreated cells and cells stably transduced with empty vector pLVX, pLVX IDH1^wt^, or pLVX IDH1^R132H^ were irradiated with a single dose of 0, 2, and 4 Gy under normoxia (21% O_2_) and hypoxia (< 0.1% O_2_), respectively. 

**Figure 1:**
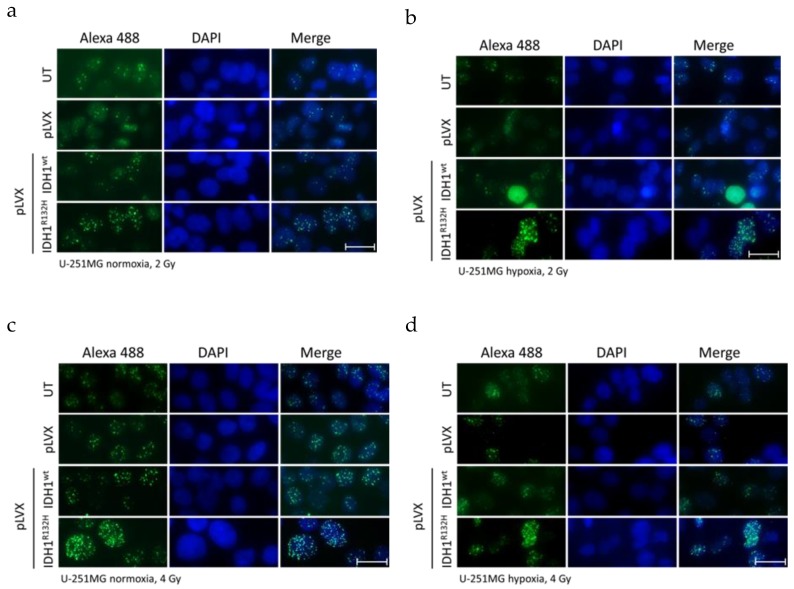
Effect of isocitrate dehydrogenase 1 (IDH1)^R132H^ gene expression on the accumulation of residual γH2AX foci after radiation in U-251MG cells. Representative immunofluorescence images of γH2AX of U-251MG cells 24 h after irradiation with 2 (**a**,**b**) and 4 Gy (**c**,**d**) under normoxia (**a**,**c**) and hypoxia (**b**,**d**). Green: γH2AX foci; blue: Cell nuclei (DAPI). n = 3 independent experiments; scale bar = 25 µm. Normoxia (21% O_2_), hypoxia (< 0.1% O_2_). UT: untreated, pLVX: cells stably transduced with empty vector, pLVX IDH1^wt^: IDH1^wt^-expressing cells, pLVX IDH1^R132H^: IDH1^R132H^-expressing cells.

**Figure 2:**
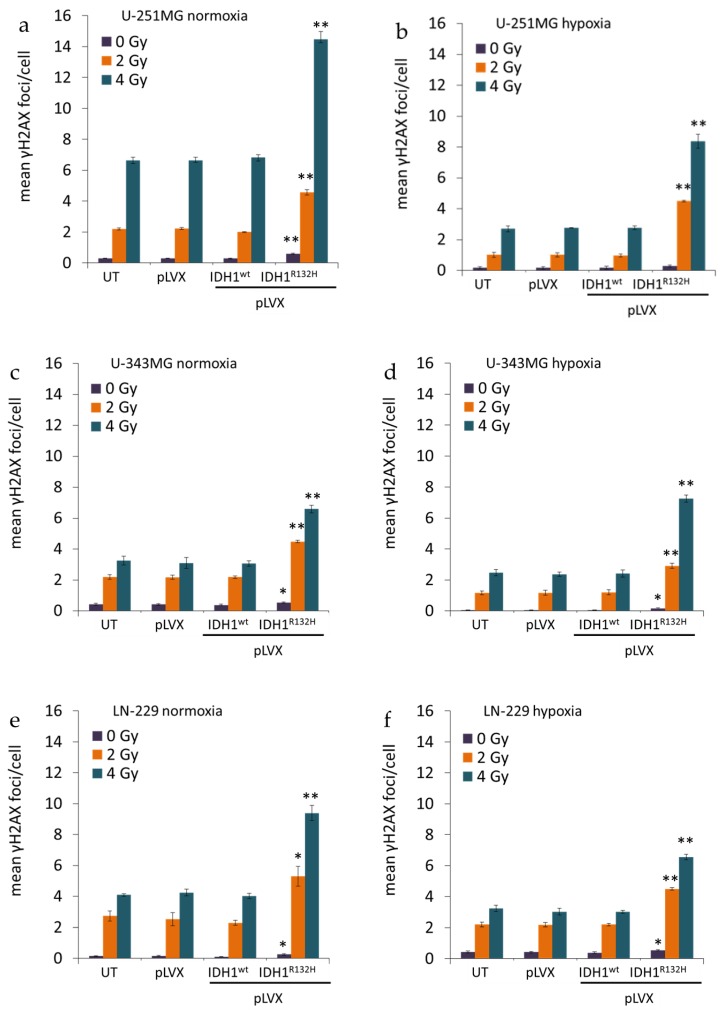
Effect of IDH1^R132H^ gene expression on the number of residual γH2AX foci after radiation in glioma cells. Mean γH2AX foci per cell nuclei of U-251MG (**a**,**b**), U-343MG (**c**,**d**), and LN-229 (**e**,**f**) cells. DNA damage was analyzed by γH2AX staining at 24 h after irradiation with 0, 2, and 4 Gy. γH2AX foci were counted manually in the nuclei of 300–400 untreated, empty vector, IDH1^wt^ or IDH1^R132H^ cells under normoxia (21% O_2_) and hypoxia (< 0.1% O_2_), respectively. Bars represent the mean values of three independent experiments. Error bars indicate standard deviations (±SD). UT: untreated, pLVX: cells stably transduced with empty vector, pLVX IDH1^wt^: IDH1^wt^-expressing cells, pLVX IDH1^R132H^: IDH1^R132H^-expressing cells; * *p* < 0.05 and ** *p* < 0.01 (compared to the respective IDH1^wt^ cells in normoxia or hypoxia).

**Figure A1:**
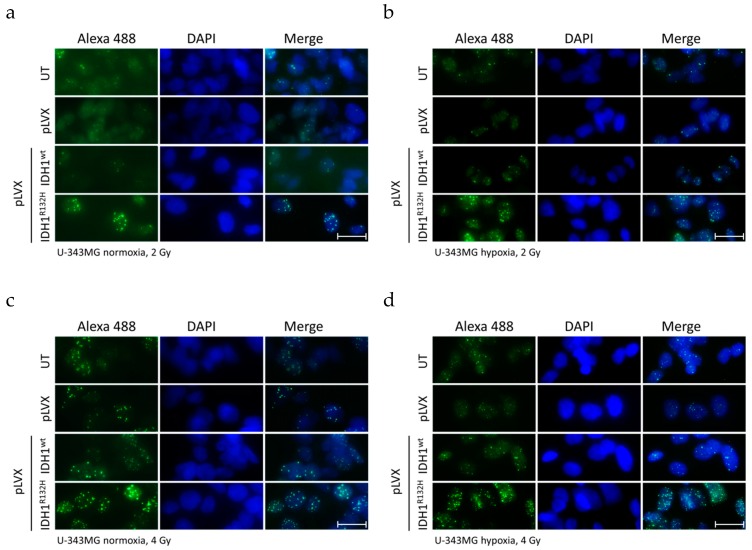
Effect of IDH1^R132H^ gene expression on the accumulation of residual γH2AX foci after radiation in U-343MG cells. Representative immunofluorescence images of γH2AX of U-343MG cells 24 h after irradiation with 2 (**a**,**b**) and 4 Gy (c, d) under normoxia (**a**,**c**) and hypoxia (**b**,**d**). Green: γH2AX foci; Blue: Cell nuclei (DAPI). n = 3 independent experiments; scale bar = 25 µm. Normoxia (21% O_2_), hypoxia (< 0.1% O_2_). UT: untreated, pLVX: cells stably transduced with empty vector, pLVX IDH1^wt^: IDH1^wt^-expressing cells, pLVX IDH1^R132H^: IDH1^R132H^-expressing cells.

**Figure A2:**
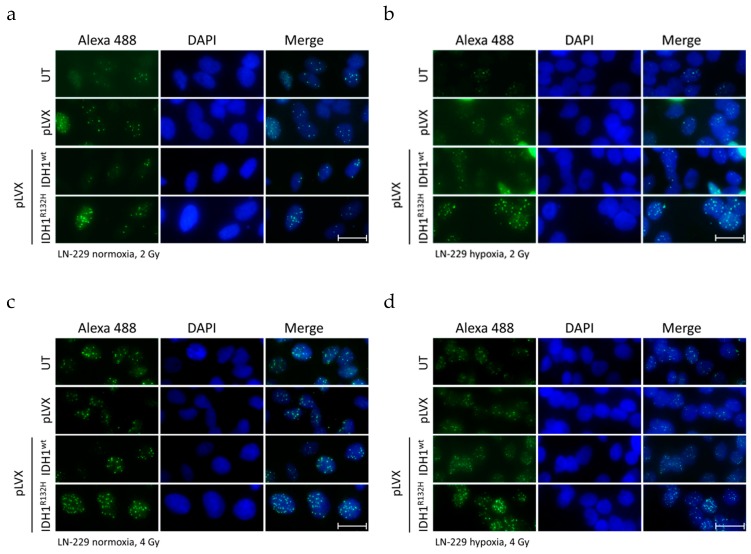
Effect of IDH1^R132H^ gene expression on the accumulation of residual γH2AX foci after radiation in LN-229 cells. Representative immunofluorescence images of γH2AX of LN-229 cells 24 h after irradiation with 2 (**a**,**b**) and 4 Gy (**c**,**d**) under normoxia (**a**,**c**) and hypoxia (**b**,**d**). Green: γH2AX foci; Blue: Cell nuclei (DAPI). n = 3 independent experiments; scale bar = 25 µm. Normoxia (21% O_2_), hypoxia (< 0.1% O_2_). UT: untreated, pLVX: cells stably transduced with empty vector, pLVX IDH1^wt^: IDH1^wt^-expressing cells, pLVX IDH1^R132H^: IDH1^R132H^-expressing cells.

**Figure A3:**
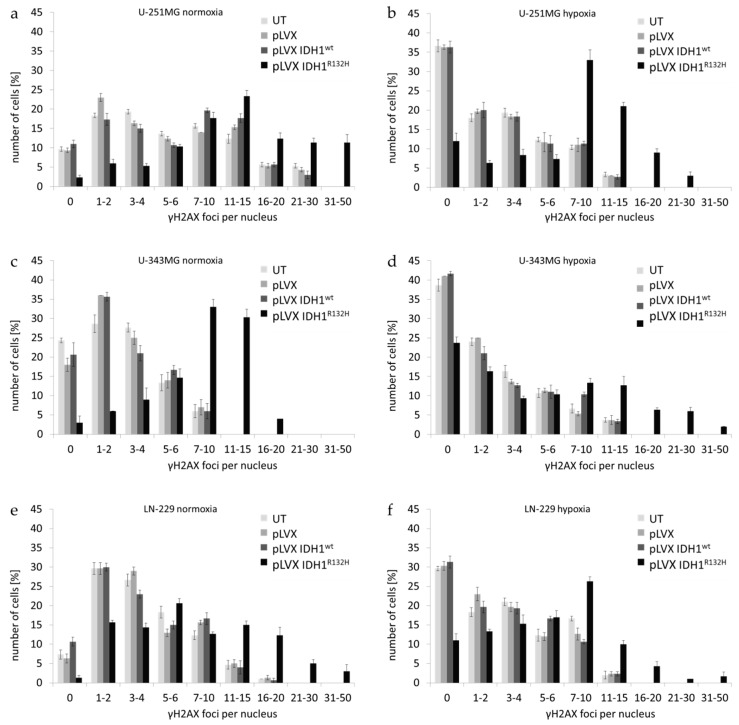
Effect of IDH1^R132H^ gene expression on the distribution of residual γH2AX foci after radiation in glioma cells. Distribution of UT, pLVX, IDH1^wt^-expressing and IDH1^R132H^-expressing cells depending on the number of foci. In U-251MG (**a**,**b**), U-343MG (**c**,**d**), and LN-229 (**e**,**f**) cells residual γH2AX foci were quantified manually as foci per nucleus of 300–400 untreated, empty vector, IDH1^wt^ or IDH1^R132H^ cells under normoxia (21% O_2_) and hypoxia (< 0.1% O_2_). γH2AX staining was achieved 24 h after radiation with 4 Gy. Bars represent the mean values of three independent experiments. Error bars indicate standard deviations (±SD). UT: untreated, pLVX: cells stably transduced with empty vector, pLVX IDH1^wt^: IDH1^wt^-expressing cells, pLVX IDH1^R132H^: IDH1^R132H^-expressing cells.

Induction of DNA DSBs and the associated cellular repair capacity was investigated by visualization (Figure 1, representative images of U-251MG; Figure A1 and Figure A2, representative images of U-343MG and LN-229) and quantitation (Figure 2 and Figure A3) of the residual γH2AX foci 24 h after radiation. Treatment with the empty vector or gene expression of IDH1^wt^ did not affect the number of γH2AX foci compared to the respective untreated U-251MG, U-343MG, and LN-229 cells in normoxia and hypoxia, respectively. 

After irradiation with 0, 2, and 4 Gy the average number of γH2AX foci per cell increased in a dose dependent manner in U-251MG, U-343MG, and LN-229 cells under normoxic and hypoxic conditions (Figure 2). Furthermore, in hypoxia γH2AX foci accumulation was decreased irrespective of the dose level in comparison to normoxic conditions in the investigated cell lines (Figure 2). Under hypoxic conditions, in untreated, empty vector and IDH1^wt^ cells, the γH2AX foci formation was up to 2.5-fold lower in U-251MG, up to 1.9-fold lower in U‑343MG and up to 1.4-fold lower in LN-229 cells compared to the respective cells under normoxic conditions (Figure 2). 

In normoxia, the non-irradiated cells gene expression of IDH1^R132H^ increased the number of γH2AX foci by 2.1-fold (*p* < 0.01) from 0.28 foci/nucleus to 0.58 foci/nucleus in U-251MG, by 1.4-fold (*p* < 0.05) from 0.38 foci/nucleus to 0.54 foci/nucleus in U-343MG cells and by 2.5-fold (*p* < 0.05) from 0.1 foci/nucleus to 0.25 foci/nucleus in LN-229 cells compared to the respective IDH1^wt^ cells (Figure 2, purple bar). Furthermore, in normoxia, after irradiation at 2 Gy gene expression of IDH1^R132H^ increased the number of γH2AX foci by 2.3-fold (*p* < 0.01) from 2 foci/nucleus to 4.6 foci/nucleus in U-251MG, by 2.0-fold (*p* < 0.01) from 2.2 foci/nucleus to 4.5 foci/nucleus in U-343MG cells and by 2.3-fold (*p* < 0.05) from 2.3 foci/nucleus to 5.3 foci/nucleus in LN-229 cells compared to the respective IDH1^wt^ cells (Figure 2, orange bar). In addition, after irradiation with 4 Gy IDH1^R132H^ cells showed an increase of γH2AX foci formation by 2.1-fold (*p* < 0.01) from 6.8 foci/nucleus to 14.5 foci/nucleus in U-251MG, by 2.1-fold (*p* < 0.01) from 3.1 foci/nucleus to 6.6 foci/nucleus in U-343MG cells and by 2.4-fold (*p* < 0.01) from 4.0 foci/nucleus to 9.4 foci/nucleus in LN-229 cells in normoxia (Figure 2, blue bar). 

Under hypoxic conditions, in the gene expression of IDH1^R132H^ increased the number of γH2AX foci by 1.7-fold (not significant) from 0.17 foci/nucleus to 0.29 foci/nucleus in U-251MG, by 3.2-fold (*p* < 0.05) from 0.05 foci/nucleus to 0.16 foci/nucleus in U-343MG cells and by 1.4-fold (*p* < 0.05) from 0.38 foci/nucleus to 0.54 foci/nucleus in LN-229 cells compared to the respective IDH1^wt^ cells (Figure 2, purple bar). In addition, under hypoxic conditions, when cells were irradiated at 2 Gy, the gene expression of IDH1^R132H^ increased the number of γH2AX foci by 4.5-fold (*p* < 0.01) from 1.0 foci/nucleus to 4.5 foci/nucleus in U-251MG, by 2.4-fold (*p* < 0.01) from 1.2 foci/nucleus to 2.9 foci/nucleus in U-343MG cells and by 2.0-fold (*p* < 0.01) from 2.2 foci/nucleus to 4.5 foci/nucleus in LN-229 cells compared to the respective IDH1^wt^ cells (Figure 2, orange bar). Furthermore, in hypoxia after irradiation at 4 Gy gene expression of IDH1^R132H^ increased the γH2AX foci formation about 3.0-fold (*p* < 0.01) from 2.8 foci/nucleus to 8.4 foci/nucleus in U‑251MG, 3.0-fold (*p* < 0.01) from 2.4 foci/nucleus to 7.3 foci/nucleus in U-343MG cells and 2.2‑fold (*p* < 0.01) from 3.0 foci/nucleus to 6.6 foci/nucleus in LN-229 cells compared to the IDH1^wt^ cells, respectively (Figure 2, blue bar).

Further, the fraction of cells in dependence of the number of residual γH2AX foci per nucleus was evaluated (Figure A3). In untreated, empty vector and IDH1^wt^ cells a higher percentage of cells with low amount of foci per nucleus was observed (Figure A3). In contrast, IDH1^R132H^-expressing cells showed an increased percentage of cells with high number of residual γH2AX foci per nucleus in normoxia and hypoxia (Figure A3).

### 2.2. Expression of IDH1^R132H^ Decreased the Amount of GSH

IDH1 is involved in a variety of cellular processes, including the glutamine metabolism and regulation of the cellular redox status via GSH [29,30,31]. Based on the decreased enzyme activity of IDH1^R132H^ and the neomorphic enzyme function, which lowers α-KG as well as NADPH levels, the GSH/GSSG ratio was measured. Different incubation times (1 h, 6 h, 24 h, and 48 h) after irradiation were analyzed in pilot experiments (data not shown). In accordance to pilot experiments and γH2AX assay, GSH/GSSG ratio was measured 24 h after irradiation with 0 or 5 Gy in an untreated, empty vector, IDH1^wt^ and IDH1^R132H^ cells of U-251MG, U-343MG, and LN-229 cells [41].

**Figure 3:**
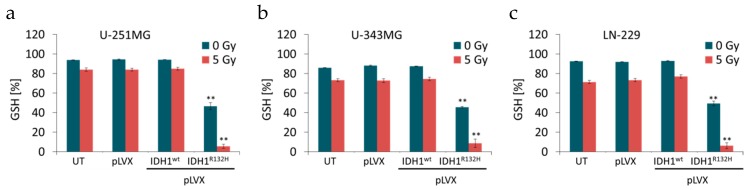
Effect of IDH1^R132H^ expression on the level of glutathione (GSH) in glioma cells. GSH level in stably transduced U-251MG (**a**), U-343MG (**b**), and LN-229 (**c**) cells after irradiation with 0 or 5 Gy. Total GSH levels (sum of reduced GSH and oxidized GSSG) were set as 100%. Bars represent the mean values of four independent experiments. Error bars indicate standard deviations (±SD). UT: untreated, pLVX: cells stably transduced with empty vector, pLVX IDH1^wt^: IDH1^wt^-expressing cells, pLVX IDH1^R132H^: IDH1^R132H^-expressing cells. ** *p* < 0.01 (compared to the respective IDH1^wt^ cells).

For evaluation of the data total GSH levels (sum of reduced GSH and oxidized GSSG) were set as 100%. Neither the treatment with the empty vector nor the gene expression of IDH1^wt^ affected the amount of reduced GSH compared to the untreated U-251MG, U-343MG, and LN-229 cells, respectively (Figure 3). On the contrary, the gene expression of IDH1^R132H^ resulted in a decreased amount of reduced GSH by 47.7% ± 4.2 (*p* < 0.01) in U-251MG, by 42.0% ± 1.04 (*p* < 0.01) in U-343MG and by 43.5% ± 2.8 (*p* < 0.01) in LN-229 cells compared to the respective non-irradiated IDH1^wt^ cells (Figure 3, blue bars). After irradiation with 5 Gy in untreated, empty vector and IDH1^wt^ cells, the level of reduced GSH was up to 10% lower in U-251MG, up to 15% lower in U‑343MG and up to 21% lower in LN-229 cells compared to non-irradiated cells, respectively (Figure 3, blue vs. red bars). Furthermore, expression of IDH1^R132H^ and irradiation with 5 Gy show an additive inhibitory effect on the amount of reduced GSH resulting in a decreased level of GSH by 79.5% ± 4.1 (*p* < 0.01) in U‑251MG, by 65.8% ± 4.2 (*p* < 0.01) in U-343MG and by 70.7% ± 5.2 (*p* < 0.01) in LN-229 cells as compared to the irradiated (5 Gy) IDH1^wt^ cells, respectively (Figure 3, red bars).

### 2.3. Expression of IDH1^R132H^ Caused a Significant Increase of Cell Stiffness

In a previous study we showed that gene expression of mutated IDH1 causes a reduced aggressiveness based on slightly decreased cell proliferation and plating efficiency, altered growth properties in 3D spheroid culture and significantly reduced cell migration [41]. In order to spread and form metastases, aggressive cancer cells with high metastatic potential might benefit from their softness and flexibility [52,53,54]. Based on these analyses we used AFM technology to quantify the relationship between several cell-specific parameters with a network analytical approach to compile the composite parameter “stiffness” (see Supplementary Methods 5) [55].

**Figure 4:**
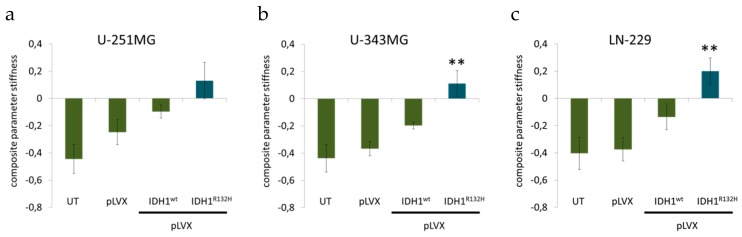
Effect of IDH1^R132H^ gene expression on the stiffness of glioma cells. Influence of IDH1^R132H^ on cell stiffness of U-251MG (**a**), U-343MG (**b**), and LN‑229 (**c**) cells was investigated using AFM technology. Bars represent the mean values of 20 single cells per measurement. Error bars indicate standard deviations (±SD). UT: untreated cells, pLVX: cells stably transduced with empty vector, pLVX IDH1^wt^: IDH1^wt^-expressing cells, pLVX IDH1^R132H^: IDH1^R132H^-expressing cells. ** *p* < 0.01 (compared to the respective IDH1^wt^ cells).

For AFM indentation measurements, single rounded cells were indented using a tip-less AFM cantilever (see Supplementary Methods 5). AFM measurements revealed that gene expression of IDH1^wt^ slightly increased the cell stiffness of glioma cell lines compared to untreated cells and empty vector cells, respectively (Figure 4). In addition, IDH1^R132H^ caused a considerable increase of cell stiffness of U-251MG (*p* = 0.1), U-343MG (*p* < 0.01), and LN-229 (*p* < 0.01) cells compared to untreated cells, empty vector cells (pLVX) and IDH1^wt^ cells (Figure 4).

### 2.4. Expression of IDH1^R132H^ Induced an Altered Organization of the Cytoskeleton

**Figure 5:**
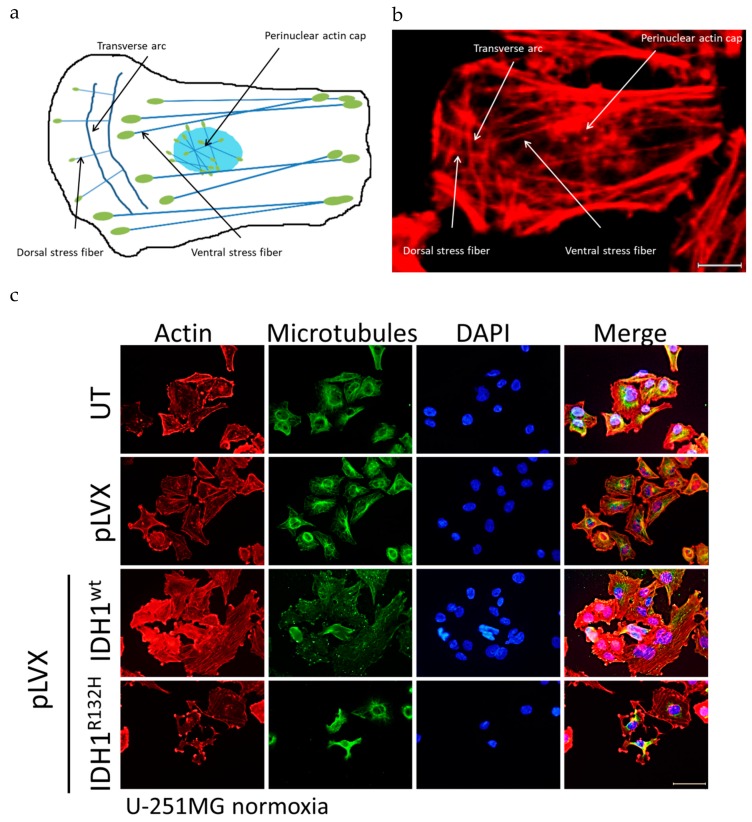
Organization of the cytoskeleton of transduced U-251MG glioma cells stably expressing IDH1^wt^ or IDH1^R312H^ protein. (**a**) Schematic representation of types of actin stress fibers. Four categories of actin stress fibers are observed: dorsal and ventral stress fibers, transverse arcs and the perinuclear actin cap. Blue: actin fibers, and green: focal adhesion. (**b**) Enlarged representative image as an example of different categories of actin stress fibers for untreated cells, empty vector cells (pLVX) and IDH1^wt^ cells; enlarged part of pLVX cells, scale bar = 10 µm (**c**) Representative immunofluorescence staining of actin stress fibers and microtubules in U-251MG cells using phalloidin-TRITC and anti-tubulin antibody. Immunofluorescence staining was performed 24 h after seeding. Cell nuclei were counterstained with DAPI. N = 3 independent experiments were performed; scale bar = 50 µm. UT: untreated, pLVX: cells stably transduced with empty vector, pLVX IDH1^wt^: IDH1^wt^-expressing cells, pLVX IDH1^R132H^: IDH1^R132H^-expressing cells.

The cytoskeleton is a meshwork of a variety of biopolymers (e.g., actin, microtubules, intermediate filaments), which are essential for dynamic functions and mechanical stability. Actin filaments assemble into diverse protrusive and contractile structures to provide force for a number of vital cellular processes including cell adhesion, morphogenesis and mechanotransduction [56]. Therefore, the actin stress fibers can be divided into at least four different categories: dorsal and ventral stress fibers, transverse arcs and the perinuclear actin cap (schematic Figure 5a) [56,57]. 

**Figure 6:**
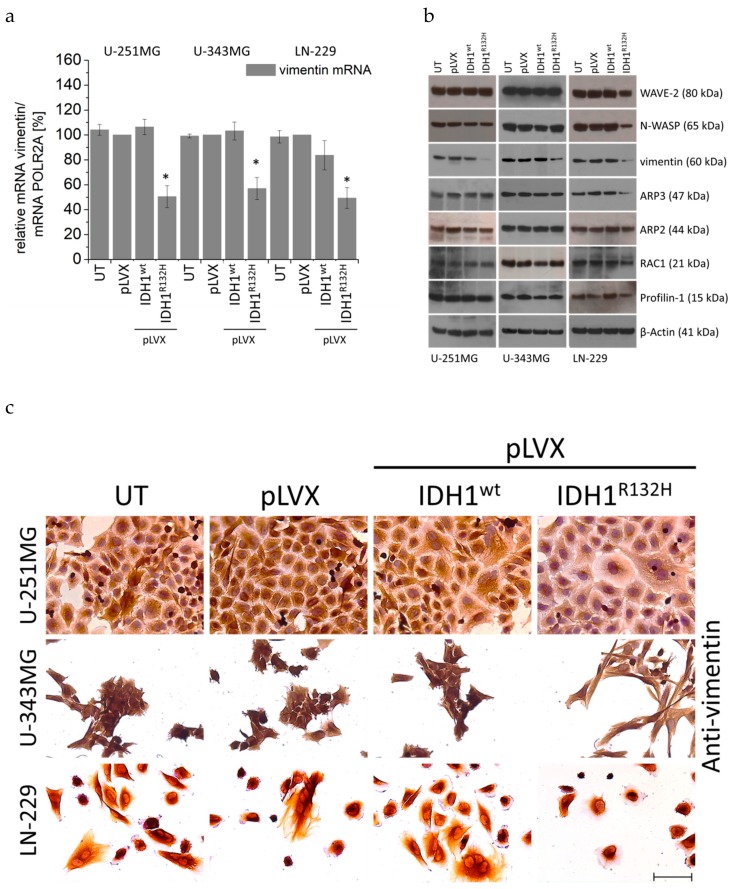
Effect of IDH1^wt^ or IDH1^R132H^ on the expression of actin-influencing proteins in glioma cells. (**a**) mRNA expression levels (qPCR) of vimentin in U-251MG, U-343MG, and LN-229 cells. Bars represent the mean values, relative to the control (empty vector pLVX set as 100%), of three independent experiments. Error bars indicate standard deviations (±SD). (**b**) Representative western blots for actin-influencing proteins of stably transduced U-251MG, U-343MG, and LN-229 cells using an Actin Nucleation and Polymerization Antibody Sampler Kit (cell signaling). n = 3 independent experiments. (**c**) Representative immunohistochemical staining of vimentin of U-251MG, U-343MG, and LN-229 cells. Cell nuclei were counterstained with 20% hematoxylin. n = 3 independent experiments; scale bar = 75 µm. UT: untreated, pLVX: cells stably transduced with empty vector, pLVX IDH1^wt^: IDH1^wt^-expressing cells, pLVX IDH1^R132H^: IDH1^R132H^-expressing cells.

**Figure A4:**
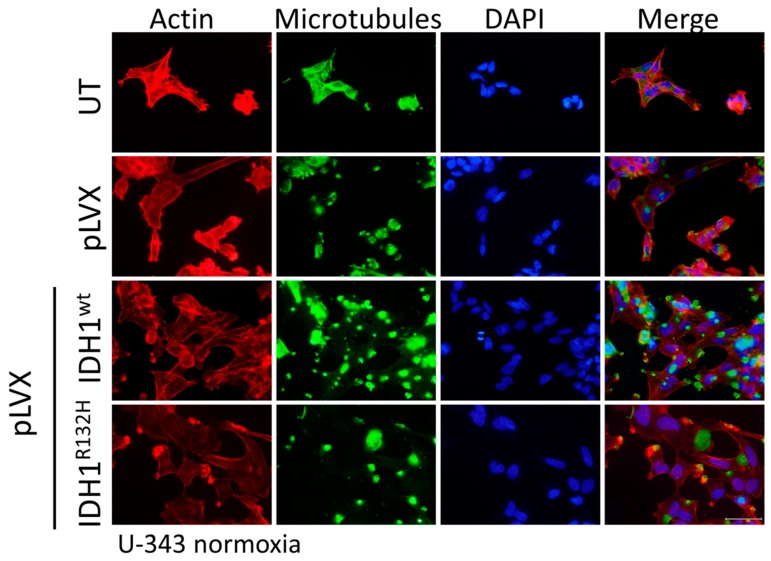
Immunofluorescence staining of transduced U-343MG glioma cells stably expressed IDH1^wt^ or IDH1^R312H^ protein. Representative immunofluorescence staining of actin stress fibers and microtubules in U-343MG cells using phalloidin-TRITC and anti-tubulin antibody. Immunofluorescence staining was achieved 24 h after seeding. Cell nuclei were counterstained with DAPI. n = 3 independent experiments; scale bar = 50 µm. UT: untreated, pLVX: cells stably transduced with empty vector, pLVX IDH1^wt^: IDH1^wt^-expressing cells, pLVX IDH1^R132H^: IDH1^R132H^-expressing cells.

**Figure A5:**
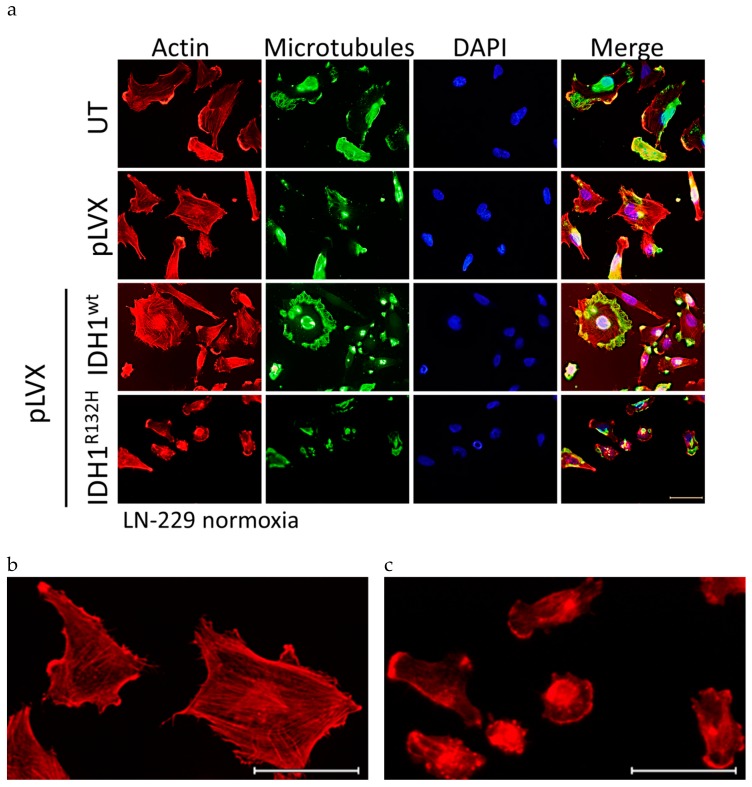
Immunofluorescence staining of transduced LN-229 glioma cells stably expressed IDH1^wt^ or IDH1^R312H^ protein. (**a**) Representative immunofluorescence staining of actin stress fibers and microtubules in LN-229 cells using phalloidin-TRITC and anti-tubulin antibody. Immunofluorescence staining was achieved 24 h after seeding. Cell nuclei were counterstained with DAPI. n = 3 independent experiments; scale bar = 50 µm. (**b**) Enlarged representative immunofluorescence staining as a representative example for untreated cells, empty vector cells (pLVX) and IDH1^wt^ cells; the enlarged part is taken from pLVX cells; scale bar = 50 µm. (**c**) Enlarged representative immunofluorescence staining as a representative example for untreated cells, empty vector cells (pLVX) and IDH1^R132H^ cells; the enlarged part is taken from IDH1^R132H^ cells; scale bar = 50 µm. UT: untreated, pLVX: cells stably transduced with empty vector, pLVX IDH1^wt^: IDH1^wt^-expressing cells, pLVX IDH1^R132H^: IDH1^R132H^-expressing cells.

Hence, immunofluorescence was applied to analyze the organization of actin stress fibers and microtubules in untreated cells, empty vector cells, and IDH1^wt^- or IDH1^R132H^-positive U-251MG, U-343MG, and LN-229 cells (Figure 5, Figure A4, Figure A5). Analysis of the stained cells showed no effect on the organization of microtubule fibers in the investigated cell lines. In addition, immunofluorescence staining indicated the presence of the four different categories of actin stress fibers in untreated cells, empty vector cells (pLVX) and IDH1^wt^ cells (enlarged and representative, Figure 5b). On the contrary, expression of IDH1^R132H^ induced an altered organization of the actin cytoskeleton of malignant glioma cells (Figure 5c, Figure A4, Figure A5). IDH1^R132H^-positive cells displayed changes in spatial distribution of actin stress fibers with fibers located in the cell periphery and thicker fibers, which have been shown to reinforce cell stiffness (Figure 5c, Figure A4, Figure A5). Furthermore, expression of IDH1^R132H^ seems to induce a smaller surface area compared to the untreated cells, empty vector or IDH1^wt^-positive cells but of LN-229 cells only (Figure A5 and Figure 6b). 

### 2.5. Expression of IDH1^R132H^ Decreased the Expression of Vimentin

To determine the effect of an expression of IDH1 mutant protein on genes which are involved in the organization of the actin cytoskeleton (actin-regulating proteins, actin-binding proteins, actin structure proteins, and intermediate filaments) qPCR, western blot analyses and immunohistochemical staining were carried out. Analyses by qPCR included actin-regulating proteins: RAC1 (Ras-related C3 botulinum toxin substrate 1), RHOA (Ras homolog gene family, member A), RHOB (Ras homolog gene family, member B), RHOC (Ras homolog gene family, member C), CDC42 (Cell division control protein 42 homolog); actin-binding proteins: Cofilin-1, Profilin-1, Fascin, Filamin, Ezrin, Moesin, alpha-actinin-1, alpha-actinin-4; actin-structure proteins: Myo1B (Myosin1B), Myo9B (Myosin9B), Myo10B (Myosin10B), Myo18A (Myosin18A), and intermediate filaments: vimentin, desmin and GFAP (glial fibrillary acidic protein). 

**Figure A6:**
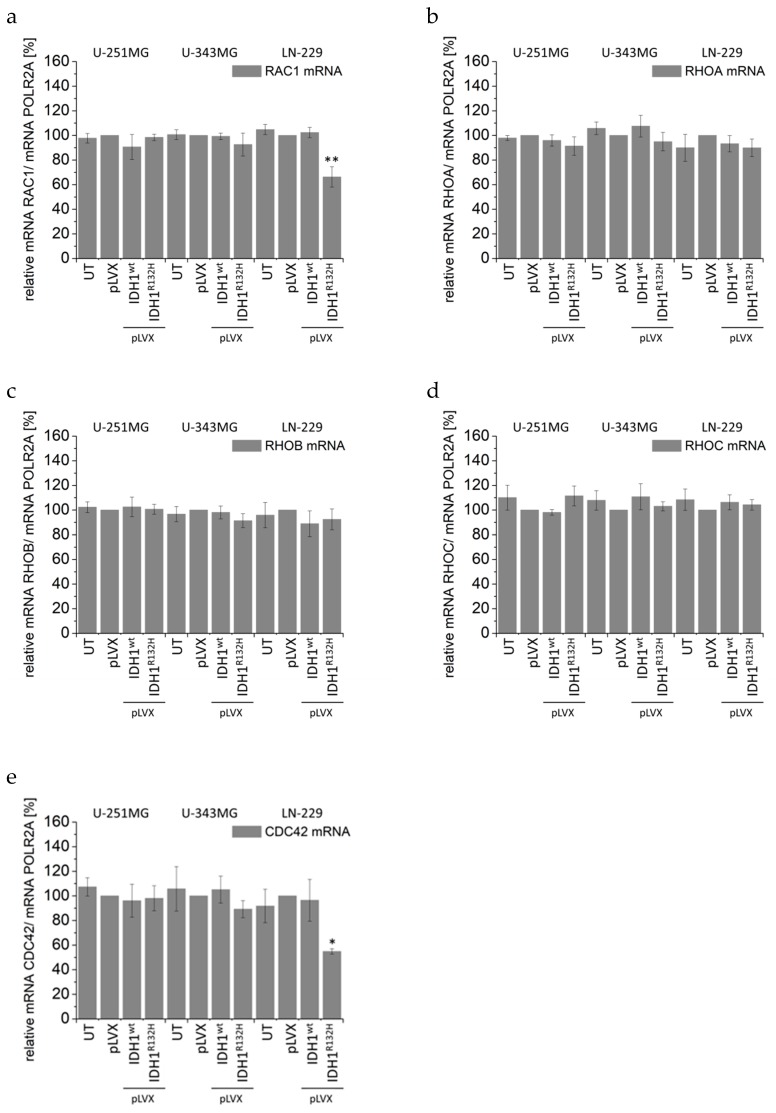
Effect of IDH1^wt^ or IDH1^R132H^ on mRNA expression of actin-regulating proteins. mRNA expression levels (qPCR) of RAC1 (**a**), RHOA (**b**), RHOB (**c**), RHOC (**d**), and CDC42 (**e**) in stably transduced U-251MG, U-343MG, and LN-229 cells. Bars represent the mean values, relative to the control (empty vector pLVX set as 100%), of three independent experiments. Error bars indicate standard deviations (±SD). UT: untreated cells, pLVX: cells stably transduced with empty vector, pLVX IDH1^wt^: IDH1^wt^-expressing cells, pLVX IDH1^R132H^: IDH1^R132H^-expressing cells. * *p* < 0.05 and ** *p* < 0.01 (compared to the respective IDH1^wt^ cells).

**Figure A7:**
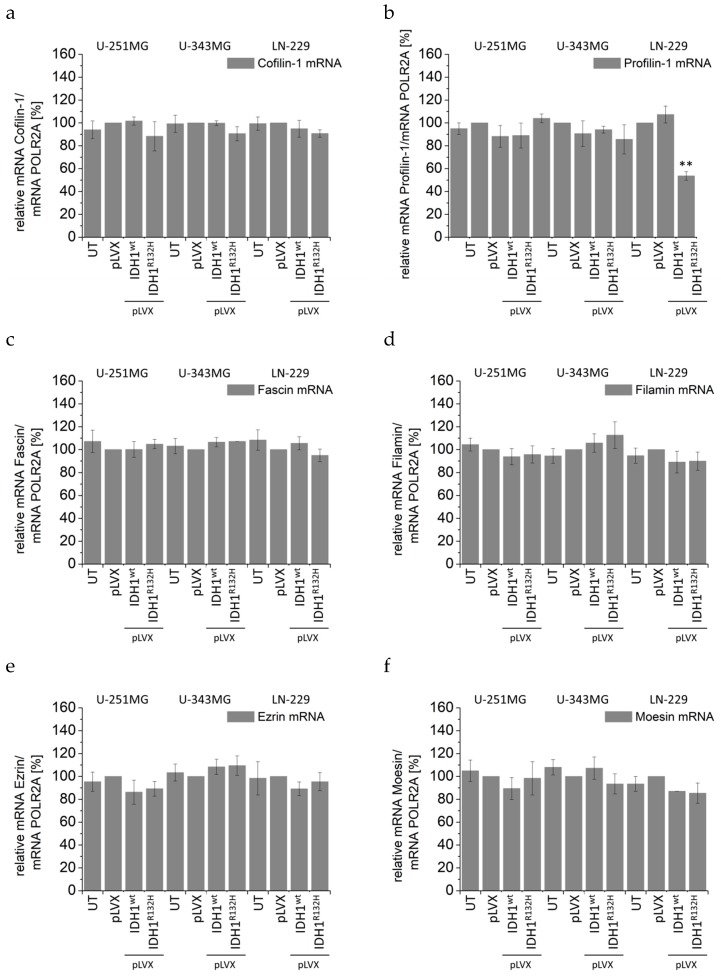
Effect of IDH1^wt^ or IDH1^R132H^ on mRNA expression of actin-binding proteins. mRNA expression levels (qPCR) of Cofilin-1 (**a**), Profilin-1 (**b**), Fascin (**c**), Filamin (**d**), Ezrin (**e**), and Moesin (**f**) in stably transduced U-251MG, U-343MG, and LN-229 cells. Bars represent the mean values, relative to the control (empty vector pLVX set as 100%), of three independent experiments. Error bars indicate standard deviations (±SD). UT: untreated cells, pLVX: cells stably transduced with empty vector, pLVX IDH1^wt^: IDH1^wt^-expressing cells, pLVX IDH1^R132H^: IDH1^R132H^-expressing cells. ** *p* < 0.01 (compared to the respective IDH1^wt^ cells).

**Figure A8:**
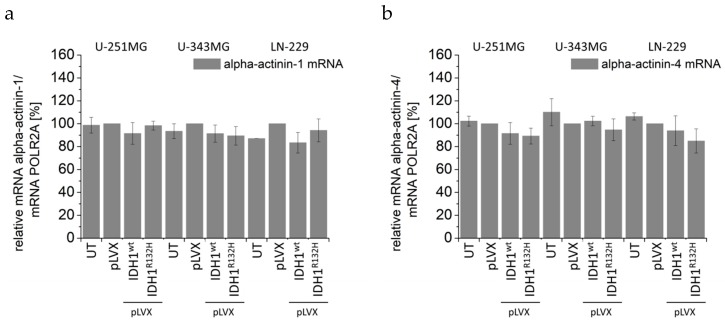
Effect of IDH1^wt^ or IDH1^R132H^ on mRNA expression of actin-binding proteins. mRNA expression levels (qPCR) of alpha-actinin-1 (**a**) and alpha-actinin-4 (**b**) in stably transduced U-251MG, U-343MG, and LN-229 cells. Bars represent the mean values, relative to the control (empty vector pLVX set as 100%), of three independent experiments. Error bars indicate standard deviations (±SD). UT: untreated cells, pLVX: cells stably transduced with empty vector, pLVX IDH1^wt^: IDH1^wt^-expressing cells, pLVX IDH1^R132H^: IDH1^R132H^-expressing cells.

**Figure A9:**
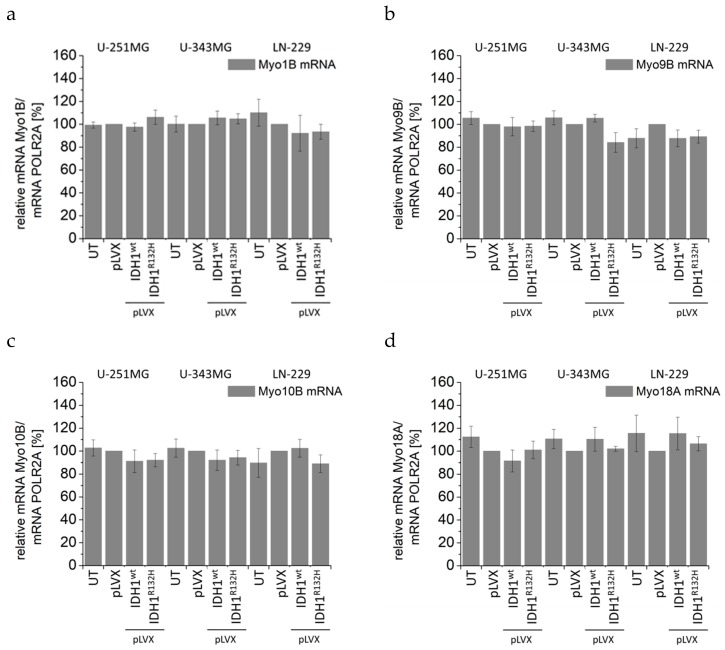
Effect of IDH1^wt^ or IDH1^R132H^ on mRNA expression of actin-structure proteins. mRNA expression levels (qPCR) of Myo1B (**a**), Myo9B (**b**), Myo10B (**c**), and Myo18A (**d**) in stably transduced U-251MG, U-343MG, and LN-229 cells. Bars represent the mean values, relative to the control (empty vector pLVX set as 100%), of three independent experiments. Error bars indicate standard deviations (±SD). UT: untreated cells, pLVX: cells stably transduced with empty vector, pLVX IDH1^wt^: IDH1^wt^-expressing cells, pLVX IDH1^R132H^: IDH1^R132H^-expressing cells.

**Figure A10:**
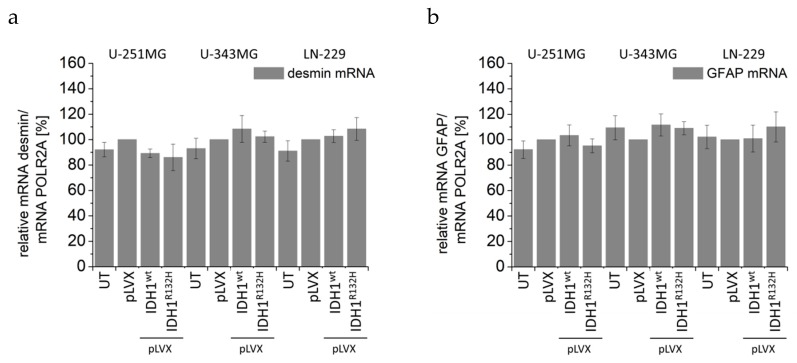
Effect of IDH1^wt^ or IDH1^R132H^ on mRNA expression of intermediate filaments. mRNA expression levels (qPCR) of desmin (**a**) and GFAP (**b**) in stably transduced U-251MG, U-343MG, and LN-229 cells. Bars represent the mean values, relative to the control (empty vector pLVX set as 100%), of three independent experiments. Error bars indicate standard deviations (±SD). UT: untreated cells, pLVX: cells stably transduced with empty vector, pLVX IDH1^wt^: IDH1^wt^-expressing cells, pLVX IDH1^R132H^: IDH1^R132H^-expressing cells.

The qPCR experiments revealed a decreased mRNA level of the intermediate filament vimentin in IDH1^R132H^-positive U-251MG (by 49.5%, *p* < 0.05), U-343MG (by 43.0%, *p* < 0.05), and LN-229 (by 50.7%, *p* < 0.05) cells (Figure 6a), whereas the other investigated genes (i.e., actin-regulating proteins see Figure A6; actin-binding proteins see Figure A7 and Figure A8; actin-structure proteins see Figure A9 and intermediate filaments see Figure A10) showed no significant alteration in their mRNA expression level in U-251MG and U-343MG cells. Furthermore, in LN-229 cells expression of IDH1^R132H^ induced a reduced mRNA level of actin-regulating proteins RAC1 (by 33.7%, *p* < 0.01), CDC42 (by 45.1%, *p* < 0.05) and of the actin-binding protein Profilin-1 (by 46.3%, *p* < 0.01), whereas no influence on the other investigated genes (RHOA, RHOB, RHOC, see Figure A6; Cofilin-1, Fascin, Filamin, Ezrin, Moesin, alpha-actinin-1, alpha-actinin-4 see Figure A7, Figure A8; Myo1B, Myo9B, Myo10B, Myo18A see Figure A9; intermediate filaments: desmin and GFAP was observed (Figure A10).

In addition, western blot analyses using an Actin Nucleation and Polymerization Antibody Sampler Kit were applied to investigate the expression of actin-influencing proteins in stably transduced U-251MG, U-343MG, and LN-229 cells (Figure 6b). The Antibody Sampler Kit includes the actin-regulating protein RAC1 (GTPase) and its targets Wiskott–Aldrich syndrome protein (N-WASP) and WASP-family verprolin-homologous protein (WAVE-2). WAVE-2, N-WASP are scaffolds that link upstream signals to the activation of the ARP2/3 complex leading to a burst of actin polymerization [58]. When activated, the Arp2/3 complex contributes the actin branched junction and thus cross-links the polymerizing actin filaments [59]. Moreover, a further protein, namely Profilin-1, was analyzed. Profilin-1 has been shown to bind ATP-bound G-actin and promotes filament elongation [60].

Western blot analyses revealed a decreased protein level of the intermediate filament vimentin in IDH1^R132H^-positive U-251MG, U-343MG, and LN-229 cells, whereas no influence on the other investigated proteins WAVE-2, N-WASP, ARP2/3, RAC1, and Profilin-1 was observed in U-251MG and U-343MG cells (Figure 6b). In accordance to the qPCR, western blot analyses confirmed the reduced expression of RAC1 and its targets WAVE-2, N-WASP in IDH1^R132H^-positive LN-229 cells (Figure 6b). Consequently, in these cells a decreased protein levels of Profilin-1 and ARP2/3, targets of WAVE-2 and N-WASP, were detected by western blot analyses (Figure 6b). 

Consistent with these findings immunohistochemical staining showed a reduction of vimentin expression in IDH1^R132H^-positive U-251MG, U-343MG, and LN-229 cells (Figure 6c).

## 3. Discussion

Diffuse gliomas, the most common type of primary brain tumors, are often diagnosed in aged adults [4,6]. On a molecular level, diffuse gliomas are characterized by a variety of genetic and epigenetic alterations. Especially the molecular marker IDH1^R132H^ has been identified as prognostic marker with major roles in tumorigenesis and the response to therapy [18,61,62]. These observations paved the way to a genotype-driven classification by including the IDH mutation as a decisive marker for glioma classification. Nowadays, these tumors are classified according to the 2016 WHO system by both histologic and molecular characteristics as IDH-mutant or IDH-wildtype astrocytomas; IDH-mutant and 1p19q-codeleted oligodendrogliomas; and IDH-mutant or IDH-wildtype glioblastomas [5]. Glioblastoma (WHO grade IV) as the most frequent and most malignant tumor in this group, i.e., is characterized by an aggressive invasiveness and a dismal prognosis with a median survival time ranging from 6 to 15 months under the current standard treatment regime, consisting of maximal surgical resection, whenever possible, followed by radiation and chemotherapy [6,25,63,64,65,66]. The heterogeneity of diffuse gliomas with respect to clinical presentation, pathology, genetic profile, and the poor response to treatment, reduces the chances for a universal treatment, particularly due to the fact that subtypes have different responses to therapy, which therefore may result in both over- or undertreatment of these tumors [3,4,38]. 

Hence, characterization of molecular tumor markers provides an opportunity to gain a deeper understanding of progression, aggressiveness and radioresistance of diffuse gliomas. In addition to the benefit in diagnosis, investigation of the IDH1 mutation represents a useful tool for the understanding of tumorigenesis and resistance to therapy of malignant diffuse gliomas. Functional studies support the development of personalized therapies as one of the main research objectives in the next years [66,67,68,69]. Based on a previous study, where the gene expression of mutated IDH1 caused an elevated radiosensitivity and a reduced aggressive biological behavior of different malignant glioma cell lines, this study dissects molecular mechanisms, underlying the influence of the most frequent IDH mutation (IDH1^R132H^) in the investigated transduced malignant glioma cells [41]. 

In clinical studies it is difficult to specifically correlate the better prognosis and prolonged overall survival of patients with IDH-mutant gliomas to the cellular behavior itself or the improved response to therapy treatment, since patients with gliomas receive standard therapy according to guidelines on management of gliomas in any case. In a previous study, the effect of IDH1 mutant protein on radiobiological behavior was carried out with U-251MG, U-343MG, and LN-229 malignant glioma cell lines. In these cells the gene expression of IDH1^R132H^ resulted in a decreased plating efficiency and enhanced induction of apoptosis under normoxia (21% O_2_) and hypoxia (< 0.1% O_2_) [26]. 

In the present work, gene expression of the mutant IDH1 effectively enhanced the radiation induced accumulation of γH2AX foci in U-251MG, U-343MG, and LN-229 malignant glioma cells irrespective of the oxygen conditions. Based on the direct correlation between the number of γH2AX foci and DSBs, quantitation of γH2AX foci formation was applied as a marker of DNA damage and repair [44,46]. Residual γH2AX foci indicate unrepaired or misrepaired DSBs, which have been shown to play a major role in radiation‑induced cell death [47]. Therefore, analyzing the induction of γH2AX foci allows for the indirect evaluation an influence of IDH1^R132H^ gene expression on the effect of radiation in U-251MG, U-343MG, and LN-229 cells. In addition, expression of IDH1^R132H^ decreased the level of reduced GSH, further expression of IDH1^R132H^ and irradiation with 5 Gy showed an additive inhibitory effect on the amount of reduced GSH. Under physiological conditions, IDH enzymes regulate a number of cellular functions and play an essential role in cellular protection as well as response to energetic and oxidative stress [29,31,36,70,71]. In addition, metabolic studies have shown that IDH activity is responsible for 65% of NADPH production capacity in the human brain. [72]. The mutated IDH1 enzyme exhibits a strongly decreased enzyme activity (loss of function) to isocitrate and NADP+ and gains an abnormal NADPH-dependent catalytic activity [33,34,35,36]. Notably, the NADPH production capacity is reduced in glioblastoma by 38% when IDH1 is mutated [36]. NADPH is required by glutathione reductase to recycle oxidized glutathione (GSSH) to reduced GSH, the major cellular ROS scavenger [33,36]. Additionally, α-KG itself functions as an antioxidant [73,74]. Thereby, it has been suggested that glioma cells expressing mutant IDH1 have a diminished antioxidant capacity and therefore may experience a subsequent loss of cytoprotection under conditions of oxidative stress [36,75,76,77]. Under these circumstances low NADPH levels might sensitize malignant gliomas for oxidative stress, amplifying the response to radiotherapy and thereby may account for the prolonged survival of patients harboring the mutations. Thus, it is possible that the enhanced radiosensitivity of U-251MG, U-343MG, and LN-229 cells is caused by the IDH1^R132H^ induced reduction of the cytoprotection against oxidative stress. Due to the key role of NADPH in the cellular antioxidation systems it has been supposed that IDH1/2 mutations may increase intracellular reactive oxygen species (ROS) by the decrease of intracellular NADPH levels [75,76,78]. Insufficient control of intracellular ROS has been associated with cellular senescence and apoptosis [79,80,81]. Consistent with these findings the gene expression of IDH1^R132H^ decreased the level of reduced GSH and consequently increased the number of residual γH2AX foci per nucleus in the non-irradiated glioma cell lines. Moreover, these effects on reduced GSH and the number of residual γH2AX foci were significantly reinforced by radiation. In accordance with these findings, IDH1 silencing of U87, A172, and U138 glioblastoma cell lines reduced levels of NADPH, deoxynucleotides and glutathione and increased their sensitivity to radiation-induced senescence [82]. In addition, a more recent study demonstrated that, even when NADPH is limiting, IDH1 mutants continue to synthesize 2-HG at the expense of other NADPH-requiring pathways that are essential for cell viability [83]. In addition, it was suggested that instead of trying to reduce 2-HG synthesis in patients, consumption of NADPH by the mutant IDH1 can be used as a metabolic weakness to sensitize tumor cells to ionizing radiation [83]. 

Among IDH-mutant gliomas, the very recent WHO classification system distinguishes three grades (II-IV) based on histopathologic features [5]. It has been widely reported in literature that with increasing malignancy gliomas exhibit intratumoral hypoxia, which has been associated with poor response to radio- or chemotherapy [7,84,85,86,87]. In the present work, gene expression of IDH1^R132H^ attenuated the hypoxia induced radioresistance of malignant glioma cells U-251MG, U-343MG and LN-229. Based on the intratumoral hypoxia of high-grade gliomas, these observations have important implications for the clinical consequences of an IDH1 mutation in these tumors. Since IDH1 is an enzyme of the tricarboxylic acid (TCA) cycle and plays a major role in energy and oxygen metabolism, it is crucial to understand how hypoxia alters the phenotype of IDH-mutant glioma cells as compared to its wildtype counterpart [88]. As already described, IDH1 catalyzes the reductive carboxylation of α-KG to isocitrate which is essential for citrate synthesis under hypoxic conditions. Likewise, cells grown under hypoxia rely almost exclusively on the reductive carboxylation of glutamine-derived α-KG for de novo lipogenesis [29,30]. The mutated IDH1 enzyme is not sufficient to catalyze the reductive carboxylation of α-KG to isocitrate, suggesting that this metabolic alteration contributes to the reduced aggressiveness of glioma cells expressing IDH1^R132H^ [89].

Based on the less aggressive phenotype of IDH1^R132H^-expressing U-251MG, U-343MG, and LN-229 cells, including a decreased cell viability, proliferation, an altered growth in 3D culture and a reduced cell migration, we analyzed cell mechanics by atomic force microscopy, the organization of the cytoskeleton as well as factors responsible for migration, stiffness and deformability of cancer cells [41]. Investigation of cell mechanics by AFM technology revealed that IDH1^R132H^ caused a considerable increase of cell stiffness compared to untreated cells, empty vector cells and IDH1^wt^ cells in all three cell lines. Cell spreading has been shown to correlate with changes of important cell functions including DNA synthesis, differentiation, cell migration, and cell stiffness [90,91]. Using AFM technology living metastatic cancer cells extracted from the pleural fluids of patients with suspected lung, breast, and pancreas cancer were more than 70% to 80% softer compared to benign mesothelial cells taken from the body cavities [52]. This study also proved that cells of different cancer types exhibit a common stiffness, whereas the stiffness of benign mesothelial cells showed a log-normal distribution. In other words, the distribution of the stiffness of tumor cells was over five times narrower than the corresponding distribution for benign mesothelial cells. In addition, nanomechanical analysis correlated well with immunohistochemical testing generally used for detecting cancer [52]. A recent study analyzed the glioma stiffness via magnetic resonance elastography (MRE). MRE demonstrated that gliomas were not only softer than normal brain, but the degree of softening was directly correlated with tumor grade and IDH1 mutation status. This means that tumors with an IDH1 mutation were significantly stiffer than those with wild-type IDH1 [92].

Based on these and other nanomechanical studies it has been suggested that cancer cells with high metastatic potential might benefit from their altered mechanical properties. Increased softness and flexibility supports spreading, as glioma cells migrate through the healthy brain tissue, mostly along anatomical structures, such as nerve fiber tracts making it necessary for cells to effectively move along the interface of different cellular structures [65,66,67,68]. Furthermore, it has also been shown that cellular stiffness of ovarian cancer cell lines and primary cells derived from ascites of patients with advanced stage ovarian cancer is inversely proportional to migration and invasion [93]. This is consistent to a study by Watanabe and coworkers who revealed that highly motile melanoma cells (B16-F10) exhibit low cell stiffness while low motile and metastatic melanoma cells (B16-F1) cells are characterized by high cell stiffness [91]. Thus, the migration activity of cancer cells seems to correlate inversely with their cellular stiffness. These findings are in accordance with own observations where gene expression of IDH1^R132H^ caused a reduced proliferation, an altered growth in 3D culture, a reduced cell migration activity and an increased cellular stiffness of U-251MG, U-343MG, and LN-229 cells. Indeed, it has already been demonstrated that gene expression of IDH1^R132H^ decreased the proliferation and migration of U-87MG (glioblastoma, grade IV) cells in normoxia [94]. In turn, this would suggest a reduced invasiveness of the IDH1^R132H^-expressing cells and may explain the better prognosis of patients with IDH1-mutant gliomas. Different studies support the relationship between the stiffness and the invasiveness of cancer cells. However, the underlying mechanism which drives cancer cells to softer mechanical characteristics and thereby to a creep deformability is not fully understood. 

In the present work, immunofluorescence was applied to analyze the organization of actin stress fibers and microtubules in untreated cells, empty vector cells, and IDH1^wt^- or IDH1^R132H^-positive cells. Immunofluorescence staining indicated the presence of the four different categories of actin stress fibers in untreated cells, empty vector cells (pLVX) and IDH1^wt^ cells. On the contrary, expression of IDH1^R132H^ induced an altered organization of the cytoskeleton of glioma cells. IDH1^R132H^-positive cells displayed changes in spatial distribution of actin stress fibers with fibers located in the cell periphery and thicker fibers, which have been shown to reinforce cell stiffness. 

Furthermore, investigation by qPCR analyses, western blot analyses and immunohistochemical staining of genes involved in the organization (actin-regulating proteins, actin-binding proteins, actin structure proteins, and intermediate filaments) of the actin cytoskeleton revealed a decreased expression of the intermediate filament vimentin in IDH1^R132H^-positive U-251MG, U-343MG, and LN-229 cells. In addition, expression of IDH1^R132H^ induced a smaller surface area and a reduced expression of RAC1 and CDC42 as well as their targets WAVE-2 and N-WASP in LN-229 cells. Consequently, in these cells a decreased protein levels of Profilin-1 and ARP2/3, targets of WAVE-2 and N-WASP, were detected by western blot analyses. CDC42 and RAC1, members of the Rho family of small G proteins, have been shown to induce actin polymerization by the Arp2/3 complex through binding to and activation of their effector proteins, N-WASP and WAVE-2, respectively [95,96,97]. The actin cytoskeleton plays a major role in the formation and function of the lamellipodia. In lamellipodia and filopodia, actin filaments are highly dynamic and directly pushes the plasma membrane forward [98]. Based on this it is likely that the reduced RAC/CDC42–WASP/WAVE–ARP2/3 signaling pathway induce a smaller surface area of LN-229 cells. Therefore, further work is required to elucidate the factors responsible for decreased surface area of LN-229 glioma cells.

Our results on cell stiffness are consistent with observations in several studies that have already shown that stiffness, deformation, and cell motility are regulated by different cellular processes. These studies have shown that actomyosin contractility, gene expression of the mesenchymal stiffness of various tumor cells [93,99,100,101,102,103,104]. Vimentin, one of the three major groups of cytoskeletal filaments, i.e., actin filaments, microtubules, and intermediate filaments has been shown to maintain mechanical cellular integrity and regulate cell stiffness [63,64,102,103]. Furthermore, vimentin was observed in a wide range of cancer types and seems to correlate with tumor aggressiveness and poor prognosis [105]. In our investigations, all three investigated cell lines showed reduced vimentin expression, an altered actin organization and increased cell stiffness. In contrast, only the cell line LN-229 showed an additional reduction of the actin-regulating proteins RAC1, CDC42 and a smaller cell surface. Therefore, it is possible that the change in vimentin expression caused the altered actin organization. Findings from different studies suggested that vimentin is indeed capable to interact and at least partly regulate focal adhesion formation and thus actin organization [106,107,108]. Jui and colleges reported a bidirectional interplay between vimentin intermediate filaments and contractile actin stress fibers. This study showed that specific actin stress fiber structures, transverse arcs, interacted with vimentin intermediate filaments and promoted their retrograde flow. Consequently, these transverse (myosin-II-containing) arcs were important for perinuclear localization of the vimentin network in cells. Further the vimentin network reciprocally restricted retrograde movement of arcs and hence controlled the width of flat lamellipodia at the leading edge of the cell [109]. In astrocytes it could furthermore be demonstrated that vimentin was necessary to maintain their polarization, indicating a possible important role for vimentin in explaining the effects of IDH1 mutations on glioma behavior [83].

In IDH mutated gliomas, elevated D-2-HG levels were induced by the gain-of-function of the mutant IDH1 protein. It is suspected that altered chromatin modifications and associated profound changes in the epigenetic status of these cells led to dysregulated gene expression [110,111,112]. The mechanism could also be a reason for the altered expression of vimentin. In the literature, clinical observations by Qi and coworkers suggest that prolonged survival of patients with IDH mutated gliomas is primarily linked to a less aggressive biological behavior assessed on the basis of preferred areas for tumorigenesis and magnetic resonance imaging (MRI) characteristics [113]. Further work will be needed to investigate possible epigenetic effects of an IDH1 mutation on genes which are involved in the organization of the actin cytoskeleton IDH1^R132H^-expressing glioma cells.

In summary, the expression of IDH1^R132H^ enhanced the radiation induced accumulation of residual γH2AX foci, decreased the amount of reduced GSH and caused a significant increase of cell stiffness of different high-grade glioma cell lines. Furthermore, the expression of IDH1^R132H^ induced an altered organization of the actin cytoskeleton, which is supposed to be an effect of the reduced expression of the intermediate filament vimentin. The enhanced radiosensitivity and increased aggressive biological behavior of IDH1^R132H^-positive malignant glioma cells are consistent with the clinical observation of a less aggressive tumor and better clinical outcome of gliomas of all WHO grades. Hence, the improved prognosis and the prolonged overall survival of glioma patients harboring the IDH1 mutation seem to be an interaction between two factors, namely the increased sensitivity to therapy and the reduced aggressiveness of these tumors. The results emphasize the important role of mutant IDH1 in treatment of patients with gliomas especially in response to radiation.

## 4. Materials and Methods

### 4.1. Generation of Constructs and Cell Culture Conditions

Cell culture conditions of the human malignant glioma cells U-251MG or LN-229 (both derived from glioblastomas, grade IV) and U-343MG cells (originate from an anaplastic astrocytoma, grade III) [42,43] were performed as previously described in detail [41]. The establishment of stable cells overexpressing IDH1^wt^ or IDH1^R132H^ was carried out as described in detail in the supplements (Supplementary Methods 1). Cell line authentication was achieved by genetic profiling using polymorphic short tandem repeat (STR) loci.

### 4.2. Hypoxia and Irradiation

Hypoxia (< 0.1% O_2_) was achieved with an Anaerocult^®^ A mini gas generation system (Merck, Darmstadt, Germany). The gas generator system is a special incubation bag that creates an anaerobic atmosphere through the presence of an Anaerocult^®^ A mini-bag containing specific components (kieselguhr, iron powder, citric acid, and sodium carbonate) that are activated by water and rapidly bind oxygen (less than 0.1% residual oxygen in the bag after 1 h). Twenty-four hours after the cells were seeded in cell numbers suitable for the experiments (depending on the area, cell density approx. 50%), the flasks containing the untreated cells or cells stably transduced (with empty vector pLVX, IDH1^wt^-, or IDH1^R132H^-expressing cells) were transferred to the Anaerocult^®^ A mini systems. After activating the Anaerocult^®^ A mini bags by wetting them with 8 mL Aqua bidest, the bags were closed with Anaeroclips^®^ and placed in a humidification incubator. The cells were incubated for another 24 h under hypoxic conditions. The detection of hypoxia was controlled by Anaerotest^®^ strips on the covers of the flasks according to the manufacturer’s protocol.

Irradiation was carried out on logarithmically growing cultures with 6 MV photons and adequate bolus material on a SIEMENS ONCOR (Erlangen, Germany) linear accelerator at a dose rate of 2 Gy/min.

### 4.3. Quantitative Real-Time PCR, Western Blot Analysis, Immunofluorescence and Immunohistochemical Staining

For real-time PCR (qPCR) analysis total RNA was extracted using TRIzol reagent (Thermo Scientific, Schwerte, Germany) as recommended by the manufacturer. DNA digestion was included by using 30 Kunitz units of RNase-free DNase in 80 μL RDD buffer (both Qiagen). RNA concentration was measured by a NanoDrop^®^ ND-1000 Spectrophotometer. cDNA was synthesized from 1 µg of RNA using RevertAid H-Minus first-strand cDNA synthesis kit (Thermo Scientific) following the manufacturer’s instructions. qPCR was performed in triplicate on a real-time PCR cycler (Rotor-Gene 6000; Qiagen, Hilden, Germany) by using Maxima SYBR Green/ROX qPCR Master Mix (Thermo Scientific). The PCR reaction conditions and a summary of all primer sequences are depicted in Table S1 of the Supplementary Material (Supplementary Methods 2; Table S1).

Protein expression was analyzed via western blot and immunofluorescence or immunohistochemical staining. The steps of protein isolation, western blot analysis and immunostaining procedures are described in detail in the Supplementary Materials (Supplementary Methods 3 and Supplementary Methods 4). The antibodies used are listed in Table S2 of the Supplement (Supplementary Methods 4; Table S2).

### 4.4. Quantification of Phospho-Histone H2AX Foci Formation

For quantification of phospho-histone H2AX (γH2AX) foci formation, untreated cells, empty vector cells and IDH1^wt^- or IDH1^R132H^-expressing cells (1 × 10^5^) were seeded in 8-well chamber slides (Thermo Scientific). After 24 h at 37 °C in normoxia, chamber slides were either kept in normoxia or incubated in Anaerocult^®^ A mini gas generator system (< 0.1% O_2_) under hypoxia. After 24 h the cells were then irradiated with 0, 2, and 4 Gy and following further incubation in normoxia or hypoxia at 37 °C for 24 h, γH2AX was analyzed via immunofluorescence staining. Details of the immunofluorescence staining and the antibodies used are described in the supplements. Quantification of γH2AX foci formation was carried out using an AxioVert 200M microscope (Carl Zeiss, Jena, Germany). The foci were counted manually in the nuclei of 300–400 untreated, empty vector, IDH1^wt^ or IDH1^R132H^ cells under normoxia and hypoxia, respectively.

### 4.5. GSH/GSSG Ratio

For measuring the GSH/GSSG ratio, cells were trypsinized, plated in 96-well plates at different cell densities ranging from 3000–5000 cells/well depending on the cell line. After 24 h the cells were irradiated with 0 or 5 Gy and GSH/GSSG Ratio was measured using GSH/GSSG-Glo™ Assay (Promega) via a luminescence signal by GENios™ plate reader (Tecan, Crailsheim, Germany) 1 h, 2 h, and 24 h after irradiation following manufactures instructions.

### 4.6. Atomic Force Microscopy

The influence of IDH1^R132H^ mutant protein expression on cell stiffness was investigated by atomic force microscopy (AFM). AFM indentation is a useful tool to analyze the mechanical properties of living cells in physiological environment at the nanoscale. For characterization of mechanical properties of single glioma cells we used two parameters namely the indentation depth (physical deformation of the cell) and the Young´s modulus [114]. These two parameters were combined to a cluster parameter called “generalized stiffness”, as described previously [55]. This parameter was used as it was demonstrated to show a strong correlation with the invasive properties of single malignant glioma cell lines [54]. Detailed information on AFM measurements can be found in Supplementary Materials (Supplementary Methods 5; Figure S1 and Figure S2).

### 4.7. Statistical Analysis

Data were analyzed by unpaired two-tailed t-tests using GraphPad Prism 6 (San Diego, CA, USA). All tests were performed using 95% confidence intervals (a p-value < 0.05 was considered significant). Results are presented as means ±SD of n = 3–4 independent experiments where * represents *p* < 0.05 and ** represents *p* < 0.01.

## 5. Conclusions

The enhanced radiosensitivity and increased cellular stiffness of IDH1^R132H^-positive glioma cells are consistent with the clinical observation of a less aggressive tumor and a prolonged survival of diffuse glioma patients harboring the IDH1 mutation. The results improve the understanding of the molecular basis of diffuse gliomas and highlight the central role of mutant IDH1 in treatment of patients with these tumors especially in response to radiation. In conclusion, detection of the genetic status of IDH1 before therapy might guide treatment decisions for patients with a less aggressive and radiosensitive IDH1-mutant diffuse glioma who should receive radiotherapy and patients with a more aggressive IDH1-wildtype diffuse gliomas who could benefit from an individually intensified therapy comprising radiotherapy and alternative medical treatment.

## Supplementary Materials

The following are available online at https://www.mdpi.com/2072-6694/11/6/889/s1, Supplementary Methods 1: Generation of constructs and stable overexpression of IDH1^wt^ and IDH1^R132H^ in glioma cell lines, Supplementary Methods 2: Real-time PCR and generation of plasmid standards, Table S1. Primers for qPCR, Supplementary Methods 3: Protein isolation and western blot analysis, Supplementary Methods 4: immunofluorescence or immunohistochemical staining, Table S2: Antibodies of western blot analysis, immunofluorescence and immunohistochemical staining, Supplementary Methods 5: Atomic force microscopy, Figure S1: Atomic force microscopy, Figure S2: Measurement principle of atomic force microscopy.
