# Supplementary material for: Radiosensitization and a Less Aggressive Phenotype of Human Malignant Glioma Cells Expressing Isocitrate Dehydrogenase 1 (IDH1) Mutant Protein: Dissecting the Mechanisms
Source: Cancers (Basel). 2019 Jun 25;11(6):889. doi: 10.3390/cancers11060889 (PMC6627228; doi:10.3390/cancers11060889)
Supplement: Supplementary file 1 [file cancers-11-00889-s001.pdf]

Supplementary Materials: Radiosensitization and a Less Aggressive Phenotype of Human Malignant Glioma Cells Expressing Isocitrate Dehydrogenase 1 (IDH1) Mutant Protein: Dissecting the Mechanisms

Jacqueline Kessler, Tim Hohmann, Antje Güttler, Marina Petrenko, Christian Ostheimer, Urszula Grabiec, Matthias Bache, Faramarz Dehghani and Dirk Vordermark

This supplementary material initially explains in greater detail the conditions of the quantitative real-time PCR (qPCR), the utilized primers are shown in supplementary Table 1. In addition, procedure of protein isolation, Western blot analysis and immunostaining is described in supplementary method 2 and 3. The antibodies are depicted in supplementary Table 2. Furthermore, atomic force microscopy (AFM) is explained in supplementary method 4. Subsequently, supplementary Figure 1 and 2 illustrate the experimental design of AFM.

Supplementary Methods 1. Generation of Constructs and Stable Overexpression of IDH1^{wt} and IDH1^{R132H} in Glioma Cell Lines

The establishment of stable cells overexpressing IDH1^{wt} or IDH1^{R132H} was performed with a lentiviral system (Clontech, Heidelberg, DE). Therefore, full-length human wildtype IDH1 coding sequence was amplified from cDNA of U-251MG cells using the following oligonucleotides IDH1 BamHI forward: 5′-GGGGATCCATGTCCAAAAAAATCAGTGGCGGTTCTGTG-3′, IDH1 XhoI reverse: 5′-GGCTCGAGTTAAAGTTTGGCCTGAGCTAGTTTGATCT-3′ and High Fidelity PCR Enzyme Mix (Thermo Scientific) according to the manufacturer's protocol. Amplification was achieved by running an initial denaturation for 5 min at 95 °C, followed by 35 cycles of denaturation for 10 sec at 95 °C, annealing for 30 sec at 66 °C, extension for 1 min at 68 °C and a final extension step for 10 min at 68 °C. The PCR product was purified using a 1 % agarose gel (1 mg agarose in 100 mL 0.5x TBE buffer and 5 μL ethidium bromide (10 mg/mL)) in 0.5x TBE buffer at a constant voltage of 200 V for 1 h. For the identification of the molecular weights of the PCR products, PageRuler™ 100 bp Plus DNA Ladder (Thermo Scientific) was used. Following electrophoresis the PCR product was cut out, isolated from the gel slice by Zymoclean™ Gel DNA Recovery Kit (Zymo Research, Irvine, US), cloned into pCR® 2.1 Vector (Thermo Scientific) to generate pCR® 2.1-IDH1^{wt} and subcloned as BamHI-XhoI (New England Biolabs) fragments into pLVX-Puro vector (kindly provided by Prof. Dr. Stefan Hüttelmaier, Institute of Molecular Medicine, Medical Faculty, Martin Luther University Halle-Wittenberg, Halle Saale, DE.). IDH1^{R132H} was obtained using the QuikChange® II XL Site-Directed Mutagenesis Kit (Stratagene, La Jolla, CA, US) following the manufacturer's instructions using IDH1^{wt} as a template and the following oligonucleotides: 5′-CCATAAGCATGATGACCTATG-3′ and 5′-CATAGGTCATCATGCTTATGG-3′. The entire *IDH1* coding region was sequenced using pLVX-Puro forward primer: 5′-CGCCATCCACGCTGTTTTGACCTCCATA-3′ and pLVX-Puro reverse primer: 5′-CCTTGGGAAAAGCGCCTCCCCTACCCGG-3′ to verify its authenticity. Sequence analyses were executed by SEQLAB Sequence Laboratories Göttingen GmbH. The NucleoBond® Plasmid Kit (Plasmid DNA Purification, NucleoBond® Xtra Midi/Maxi; Clontech) was utilized for the extraction or purification of plasmid DNA. A second generation plasmid packaging system with three plasmids was used for virus production, including the envelope encoding plasmid pMD2.G and the packaging plasmid psPAX2 (addgene, Cambridge, USA). The empty vector (10 μg) pLVX-Puro (clontech) or pLVX encoding IDH1^{wt} or IDH1^{R132H} were co-transfected with 10 μg pMD2.G and 5 μg psPAX2 into HEK293T cells using the calcium chloride DNA precipitation method.

The precipitate was formed by adding the plasmids to 0.5 mL sterile buffered H2O and 0.5 mL 2× HEPES buffered saline solution (280 mM sodium chloride (NaCl), 50 mM HEPES, 1.5 mM sodium hydrogen phosphate (Na2HPO4), pH 7.0). Subsequently, 60 μL 2.5 M calcium chloride ($CaCl_2$;

Sigma-Aldrich) was added with gentle movement of the tube. The solution was incubated for 20 min at room temperature (RT) and then added to the cells drop by drop. Twelve hours later, the medium was replaced by fresh cell culture medium. The viral supernatant was harvested 48 h after transfection and purified by centrifugation at 1,500 rpm for 5 min at RT followed by filtration through a 0.45 µm pore cellulose acetate filter (Sartorius). Glioma cells with different degrees of malignancy were transduced with empty vector (pLVX) or with pLVX encoding $IDH1^{wt}$ or $IDH1^{R132H}$ using 1 mL of the viral suspension, respectively. U-251MG (grade IV), U-343MG (grade III) and LN-229 (grade IV) stable cell lines (polyclonal) with empty vector (pLVX) or overexpressing $IDH1^{wt}$ or $IDH1^{R132H}$ were selected using 1.5 µg/mL puromycin (Sigma-Aldrich) from 48 h post transfection. Glioma cells with varying degrees of malignancy were transduced with empty vectors (pLVX) or with pLVX encoding $IDH1^{wt}$ or $IDH1^{R132H}$ with 1 mL each of the virus suspension. 48 h after transfection, U-251MG (grade IV), U-343MG (grade III) and LN-229 (grade IV) stable cell lines (polyclonal) with empty vector (pLVX) or overexpressing $IDH1^{wt}$ or $IDH1^{R132H}$ were selected using 1.5 µg/mL puromycin (Sigma-Aldrich).

Subsequently, qPCR analyses showed high IDH1 mRNA levels in pLVX $IDH1^{wt}$ and $IDH1^{R132H}$ transduced U-251MG, LN-229 and U-343MG glioma cells under both normoxic and hypoxic conditions. In addition, Western blot analyses confirmed the overexpression of IDH1wt or IDH1R132H protein in stably transduced U-251MG, U-343MG and LN 229 glioma cells under normoxic or hypoxic conditions. Immunofluorescence and immunohistochemical staining with anti-human IDH1 and mutant-specific anti-IDH1R132H antibodies (both obtained from Dianova, Hamburg, Germany) confirmed overexpression and diffuse cytoplasmic distribution of the $IDH1^{wt}$ or $IDH1^{R132H}$ protein in the investigated glioma cell lines under normoxic and hypoxic conditions [1].

Supplementary Methods 2. Real-Time PCR and Generation of Plasmid Standards

qPCR was performed by mixing 6.5 µL of SYBR Green mix, 0.25 µL of each primer (20 µM), 1 µL of cDNA and 7 µL of water. Reactions were denatured for 15 min at 95 °C, followed by 40 cycles of denaturation for 30 sec at 95 °C, annealing for 30 sec at 60 °C, extension for 30 sec at 72 °C, a final extension step for 30 sec at 60 °C and a melting curve program (65 °C-95 °C with a heating rate of 0.2 °C/s). For normalization, *Homo sapiens polymerase (RNA) II polypeptide A* (*POLR2A*) was used as housekeeping gene. The primers for the amplification step were synthesized by Sigma-Aldrich. A summary of all primer sequences used in this manuscript is provided in supplementary Table 1. DNA plasmid standard curve was performed using TA Cloning® Kit (Thermo Scientific) to determine the absolute quantity (copy numbers, ng, mRNA) of a specific gene. The respective plasmid standard was synthesized by amplifying the PCR product from cDNA of the glioma cells using HotStarTaq DNA Polymerase (Qiagen), gel-purification by Zymoclean™ Gel DNA Recovery Kit (Zymo Research, Irvine, US) and ligation into pCR® 2.1 vector via T4 DNA Ligase (Thermo Scientific). The constructs were transformed into One Shot®TOP10 Chemically Competent *E.coli* (Thermo Scientific) and 50-200 µL from each transformation was spread on LB Agar plates (Thermo Scientific) containing 5-Bromo-4-chloro-3-indolyl-ß-D-galactosid (X-Gal; Sigma-Aldrich) as well as 100 µg/µL ampicillin (Carl Roth) and further a blue-white screening for inserts was performed. According to the manufacturer's instructions white colonies were picked, grown overnight at 37 °C in LB Broth Base (Thermo Scientific) containing 100 µg/µL ampicillin and plasmid isolation was subsequently assessed by ZR Plasmid Miniprep™ Classic Kit (Zymo). The entire gene fragment region was sequenced using M13 forward primer or T7 promotor primer to verify its authenticity. A detailed description of the individual steps of the plasmid standard synthesis can be found in the manufacturer's protocol of the TA Cloning® Kit (Thermo Scientific).

Table S1. Primers for qPCR. Primer sequences and the localization of the primer binding side in the corresponding mRNA transcript. For normalization, RNA polymerase 2A (POLR2A) was used as housekeeping gene.

Gene	Primer	Sequence 5′→3′	Orientation	Localization
POLR2A	POLR2A fw	CTTGCCCCGTGCCATGCAGA	sense	1358-1377

Gene	Primer	Sequence 5′→3′	Orientation	Localization
	POLR2A rev	CTTGCGACCTTGACCATCTT	antisense	1421-1440
IDH1	IDH1 fw	CGGTCTTCAGAGAAGCCATT	sense	551-570
	IDH1 rev	AACACCACCACCTTCTTC	antisense	751-768
RAC1	RAC1 fw	AGGCCATCAAGTGTGTGGTG	sense	246-265
	RAC1 rv	GTTTGCGGATAGGATAGGGGG	antisense	445-465
RHOA	RHOA fw	GTCCACGGTCTGGTCTTCAG	sense	176-195
	RHOA rv	GCCATTGCTCAGGCAACGAA	antisense	266-285
RHOB	RHOB fw	CAGTAAGGACGAGTTCCCCG	sense	470-489
	RHOB rv	GTCCACCGAGAAGCACATGA	antisense	637-656
RHOC	RHOC fw	ATTCCTGAGAAGTGGACCCCA	sense	685-705
	RHOC rv	ATCTCAAACACCTCCCGCAC	antisense	901-920
CDC42	CDC42 fw	ATGACAGATTACGACCGCTGA	sense	357-377
	CDC42 rv	GGAGTCTTTGGACAGTGGTGA	antisense	472-492
CFL-1	Cofilin fw	GTGCCCTCTCCTTTTCGTTTC	sense	207-227
	Cofilin rv	CTTCACCTCCTCTGGCGTTG	antisense	305-324
Profilin-1	Profilin-1 fw	ACGCCTACATCGACAACCTC	sense	706-725
	Profilin-1 rv	CCCAAGTGTCAGCCCATTCA	antisense	874-893
VIM	vimentin fw	GGACCAGCTAACCAACGACA	sense	896-915
	vimentin rv	AAGGTCAAGACGTGCCAGAG	antisense	1054-1073
ACTN1	alpha actinin 1 fw	GCGAGGCAAGATGAGAGTGC	sense	553-572
	alpha actinin 1 rv	TCTTCACATTCCCATCCACGA	antisense	654-674
ACTN4	alpha actinin 4 fw	GTTACCTAAGCCGGAGCGG	sense	415-433
	alpha actinin 4 rv	AGGGTCATCTTTGCGTTGCC	antisense	539-558
CRYAB	αβ-crystallin fw	CGCCTGGAGAAGGACAGGTT	sense	512-531
	αβ-crystallin rv	GGTGAGAGGGTCTACATCAGC	antisense	683-703
Desmin	Desmin fw	CAACAAGAACAACGACGCCC	sense	1007-1026
	Desmin rv	TCACTGGCAAATCGGTCCTC	antisense	1143-1162
FSCN1	Fascin 1 fw	AACCGCCCCATCATCGTGTT	sense	1276-1295
	Fascin 1 rv	AGTCCACAGGAGTGTCGCC	antisense	1465-1483
GFAP	GFAP fw	GAAGCAGATGAAGCCACCCT	sense	589-608
	GFAP rv	TCATACTGCGTGCGGATCTC	antisense	775-794
FLNA	Filamin A fw	CGAGGTGACGGGGACTCATA	sense	1293-1312
	Filamin A rv	AGGTGGTCTTGTTGGCGATG	antisense	1437-1456
EZR	Ezrin fw	GTGGTACTTTGGCCTCCACTA	sense	341-361
	Ezrin rv	ACTTGGCCCGGAACTTGAAC	antisense	446-465
MSN	moesin fw	CCCCTGGAGTGAAATCAGGAA	sense	918-938
	moesin rv	CGGGGAGCATAGAAGACGAA	antisense	997-1016
MYO10B	MYO10 fw	AGGACTTTCCACCTGATTGC	sense	4324-4343
	MYO10 rv	CGTGGACCTGACTCAGCA	antisense	4379-4396
MYO9B	MYO9B fw	CAACCAGCACATCTTCAAGC	sense	1796-1815
	MYO9B rv	TGTTGTGCCACGTGATCC	antisense	1843-1860
MYO18A	MYO18A fw	GGACATGGTGACAAAGTATCAGAA	sense	6017-6040
	MYO18A rv	TTTGACAACCAGGACTTGACC	antisense	6104-6124
MYO1B	MYO1B fw	GGGCTTACTGGCTTGGATCT	sense	2702-2721
	MYO1B rv	ACAGCAACTGCATGCTTACG	antisense	2764-2783
M13	M13 fw	GTAAAACGACGGCCAG		
T7	T7	TAATACGACTCACTATAGGG		

Supplementary Methods 3. Protein Isolation and Western Blot Analysis

For protein isolation cells were washed twice with ice-cold PBS, harvested using a cell scraper and lysed with RIPA buffer containing Tris-HCl (50 mM, pH 8.0), 200 mM NaCl, EDTA (1 mM; AppliChem), ethylene glycol tetraacetic acid (EGTA, 1 mM; Carl Roth), 1 % TritonX-100 (Carl Roth), 0.25 % deoxycholate (AppliChem), Protease-Inhibitor-Cocktail (1:100; Sigma-Aldrich) and Halt™ Phosphatase Inhibitor Cocktail (1:100; Thermo Scientific). The lysates were incubated for 20 min on ice and centrifuged at 10,000 g for 10 min at 4 °C. The supernatants were collected and protein

concentrations were measured on a NanoDrop® ND-1000 Spectrophotometer via the Bio-Rad Protein-Assay (Bradford reagent) at 595 nm according to the manufacturer's instructions.

Equivalent amounts of protein (30 µg) were diluted in NuPAGE® LDS Sample Buffer (4x) containing NuPAGE® Sample Reducing Agent (10x) (Thermo Scientific) and denatured for 5 min at 95 °C. Protein samples and Protein Ladder (PageRuler™ Plus Prestained Protein Ladder; Thermo Scientific) were loaded on 4-12 % Bis-Tris Mini Gels and separated in NuPAGE® MES SDS running buffer (20x) using a mini-cell electrophoresis system (XCell SureLock™; Thermo Scientific) at a constant voltage of 200 V for 1 h. Proteins were transferred to a 0.45 µm PVDF membrane (Millipore, Schwalbach, DE) by tank electroblotting (Bio Rad) at 150 V for 2.5 h in transfer buffer containing 50 mL NuPAGE® Transfer Buffer (20x) (Thermo Scientific), 850 mL aqua bidest. and 100 mL methanol (Sigma-Aldrich).

After blocking the non-specific binding sites with 10 % milk powder (Carl Roth) in 0.1 M TBST for 1 h at room temperature (RT), the membranes were incubated with the primary antibodies at appropriate dilutions in TBST with 10 % milk powder or TBST with 5 % BSA (in dependence of the antibody) overnight at 4 °C. Subsequently, staining was performed for 1 h at RT with a suitable secondary horseradish peroxidase-conjugated antibody in TBST with 10 % milk powder. Details of the utilized antibodies are described in supplementary Table 2. The protein-antibody complexes were detected by enhanced chemiluminescence using ECL™ Western Blotting Detection Reagent or ECL™ Prime Western Blotting Detection Reagent (both GE Healthcare, Chalfont St Giles, UK). In a darkroom, the X-ray films (Thermo Scientific) were exposed to the emitted light signal and developed by successive immersion in developing solution (Nordenta, Hamburg, DE), water, fixer solution (Nordenta) and water.

Supplementary Methods 4. Immunofluorescence or Immunohistochemical Staining

Immunofluorescence analysis was performed to investigate the effect of $IDH1^{R132H}$ on γH2AX foci formation or on the organization of actin fibers in glioma cells. Cells were grown for 24 h in 8-well chamber slides (Thermo Scientific) at a density of 1.5×10^4 cells/well, fixed with 4 % paraformaldehyde (Sigma-Aldrich) for 20 min, permeabilized with 0.5 % Triton X-100/PBS for 10 min at RT. After blocking for 1 h with 1 % BSA (Promega) in PBS, the cells were incubated overnight at 4 °C with primary antibodies (P-Histone H2AX, β-Tubulin) and subsequently for 1 h at RT in the dark with suitable secondary antibodies (anti-rabbit Alexa 488-labeled or anti-mouse Alexa 488-labeled). Immunofluorescence counterstaining of the cell nuclei was performed with 4′,6-diamidino-2-phenylindole (DAPI, 0.25 µg/µL in PBS; Carl Roth) for 5 min in the dark at RT. The slides were then washed again with PBS, air-dried and mounted with ProLong®Gold Antifade Reagent (Thermo Scientific). Details of the used antibodies are listed in Table S2.

Actin filaments (F actin) were analyzed via phalloidin (Tetramethylrhodamine B isothiocyanate; Sigma-Aldrich) staining. Phalloidin, a fungal toxin isolated from the poisonous mushroom *Amanita phalloides*, has been found to bind only to polymeric and oligomeric forms of actin, and not to monomeric actin. For F actin analysis cells were grown for 24 h in 8-well chamber slides at a density of $2{,}5 \times 10^4$ cells/well, fixed with 4 % paraformaldehyde for 20 min and permeabilized with 0.5 % Triton X-100/PBS for 10 min at RT. After blocking for 1 h with 1 % BSA in PBS, the cells were incubated for 1 h with phalloidin (10 µg/mL) in the dark at RT and subsequently counterstained with DAPI. The slides were then washed again with PBS, air-dried and mounted with ProLong®Gold Antifade Reagent. Fluorescence conjugates of phalloidin and actin filaments were imaged using an AxioVert 200M microscope (Carl Zeiss, Jena, Germany).

Vimentin gene expression patterns in untreated, empty vector, $IDH1^{wt}$ or $IDH1^{R132H}$ glioma cells were investigated by immunohistochemical staining. Cells were grown for 24 h in 8-well chamber slides at a density of 1.5×10^4 cells/well were fixed with 4 % paraformaldehyde. The cells were then permeabilized with 0.5 % Triton X-100/PBS for 10 min at RT. After blocking for 10 min in Antibody Diluent (ZYTOMED Systems, Berlin, DE) the cells were incubated overnight at 4 °C with primary antibodies (supplementary Table 2) and subsequently stained via Dako REAL™ Detection System, Peroxidase/DAB+, Rabbit/Mouse (Dako, Hamburg, DE) according to the manufacturer's

instructions. After staining the target antigens, the cell nuclei were counterstained with 20 % hematoxylin in aqua bidest. for 5 min, washed with water, dehydrated in a graded ethanol (Carl Roth) series (50–100%), air-dried and mounted in Eukitt® mounting medium (Sigma-Aldrich).

Table S2. Antibodies of Western blot analysis, immunofluorescence and immunohistochemical staining. Primary antibodies used for Western blot (WB) analysis, immunofluorescence (IF) or immunohistochemical staining (IHC) and the corresponding secondary antibodies utilized for immunostaining.

Antibody	Source	Dilution		Company
		WB	IF/IHC	
IDH1wt DIA-W09	rat	1:1000		Dianova, Hamburg, Germany
IDH1R132H DIA-H09	mouse	1:1000		Dianova, Hamburg, Germany
WAVE 2 (Kit)	rabbit	1:1000		Cell Signaling, Danvers, MA, US
N-WASP (Kit)	rabbit	1:1000		Cell Signaling, Danvers, MA, US
ARP2 (Kit)	rabbit	1:1000		Cell Signaling, Danvers, MA, US
ARP3 (Kit)	rabbit	1:1000		Cell Signaling, Danvers, MA, US
RAC1 (Kit)	rabbit	1:2000		Cell Signaling, Danvers, MA, US
Phospho-RAC1 (Kit)	rabbit	1:1000		Cell Signaling, Danvers, MA, US
Profilin-1 (Kit)	rabbit	1:2000		Cell Signaling, Danvers, MA, US
Vimentin	rabbit	1:1000	1:100	Cell Signaling, Danvers, MA, US
P-Histone H2AX (S139)	rabbit	-	1:400	Cell Signaling, Danvers, MA, US
β-Tubulin	mouse	-	1:200	Sigma-Aldrich, Germany
anti-β-actin	mouse	1:5000		Sigma-Aldrich, Germany
anti-mouse HRP-conjugated	rabbit	1:1000		DAKO, Hamburg, Germany
anti-rat HRP-conjugated	goat	1:1000		Santa Cruz, Santa Cruz, CA, USA
anti-rabbit HRP-conjugated	goat	1:1000		DAKO, Hamburg, Germany
anti-mouse Alexa 488-labeled	goat	-	1:100	Invitrogen, Karlsruhe, Germany
anti-rabbit Alexa 488-labeled	goat	-	1:400	Invitrogen, Karlsruhe, Germany

Supplementary Methods 5. Atomic Force Microscopy

Cells were plated with 5x10^5 cells/flask in 75 cm^2 flasks, cultured under normoxic conditions for 48 h, trypsinized to induce detachment and centrifuged at 1000 g for 5 min. Cells were resuspended in 1 mL cell culture medium, plated on a 6 cm culture dish and incubated for 15 min allowing the cells to adhere slightly to the surface. For AFM indentation measurements a lithium nitride tip-less cantilever (Nanoworld, Arrow-TL2) tilted by 10° was used for indentation (Figure S1). To analyze the cellular mechanical properties, single cells were indented with a force of 3 nN (Figure S2). Five force curves per rounded cell were recorded. The Young´s modulus *E* (elastic modulus,) was estimated by fitting force curves with the Hertz model:

$$F = \frac{4}{3}\frac{E}{1-v^2}\sqrt{R\delta_0{}^3} \quad (1)$$

where F is force detected, E is the Young´s modulus, R is the radius of the cell, ν is the Poisson´s ratio and δ_0 is the central indentation [2]. In general, Poisson´s ratios between 0.3 for soft tissue to 0.5 for an incompressible material are used. The best-match of experimental and modelling data has been reported when cells are treated as almost incompressible. Thus a Poisson´s ratio of 0.47 was applied in the present study [3].

Furthermore, additional physical measurements carried out (indentation, normalized adhesion energy, jump energy, total adhesion energy, minimal force, jump force, slope of approach curve, cell radius, Young´s modulus and jump number) to quantify the relationship between several cell-specific parameters based on a network analytical approach to compile the nondimensional composite parameter "stiffness" as a weighted sum [4].

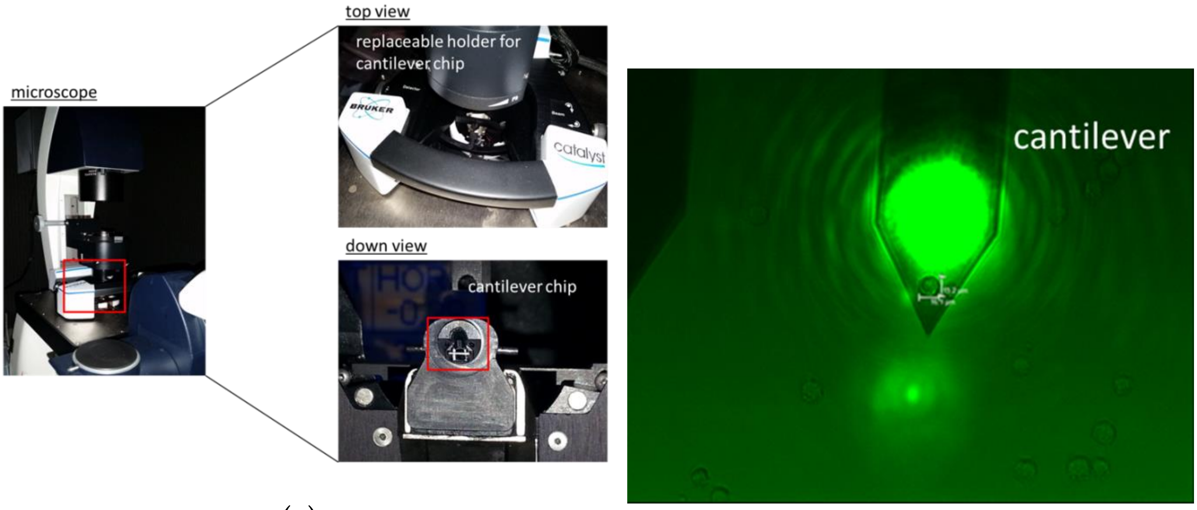

(**a**) (**b**)

Figure S1. Atomic force microscopy. (**a**) Cantilever based optical microscope. Cantilever chip was mounted on a chip holder, which is placed on the stage of an inverted optical microscope. (**b**) Microscopic image of an indentation of a single cell by the cantilever. For each cell line (U-251MG, U-343MG and LN-229) 20 single cells of untreated, empty vector cells, IDH1^{wt} or IDH1^{R132H} cells were analyzed, respectively. AFM measurements were carried out in the Core Facility Imaging, Martin Luther University Halle-Wittenberg, Halle Saale, Germany.

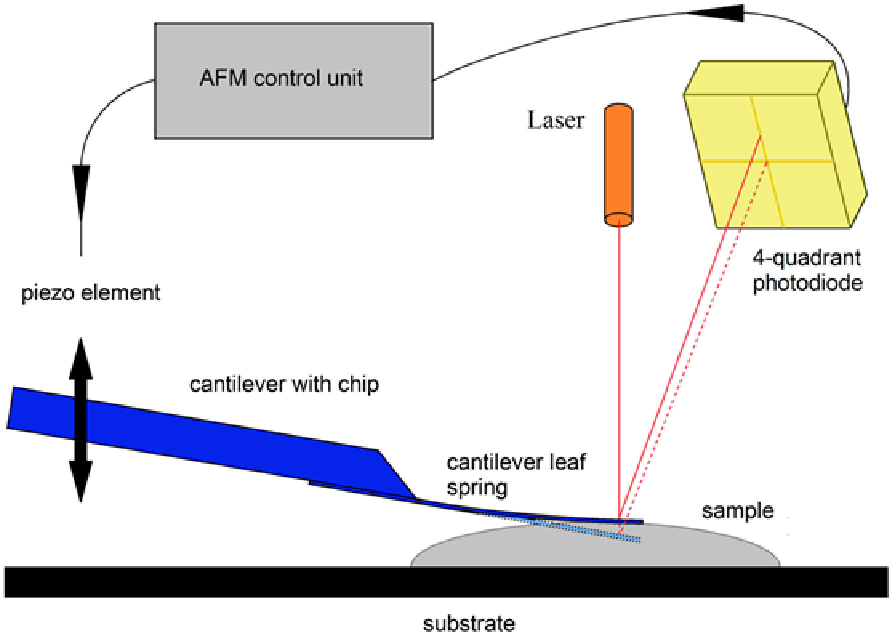

Figure S2. Measurement principle of atomic force microscopy. AFM cantilever (blue) is positioned above a cell (grey) and gently pressed with a force of 3 nN onto the cell. The laser (red line) is reflected off the back of the cantilever and the deflection is detected using a 4-quadrant-photodiode. The laser spot was initially positioned in the middle of the 4-quadrant-photodiode. Deformation of the cantilever occurs during indentation on a single cell; thereby deflection on the cantilever was detected as a change in output voltage.

References

1. Kessler, J.; Güttler, A.; Wichmann, H.; Rot, S.; Kappler, M.; Bache, M.; Vordermark, D. IDH1(R132H) mutation causes a less aggressive phenotype and radiosensitizes human malignant glioma cells independent of the oxygenation status. *Radiother. Oncol.* **2015**, *116*, 381–387, doi:10.1016/j.radonc.2015.08.007.
2. Hertz, H. Ueber die Berührung fester elastischer Körper. *Journal für die reine und angewandte Mathematik* **1882**, *92*,156–171.
3. Mahaffy, R.E.; Park, S.; Gerde, E.; Käs, J.; Shih, C.K. Quantitative Analysis of the Viscoelastic Properties of Thin Regions of Fibroblasts Using Atomic Force Microscopy. *Biophys. J.* **2004**, *86*, 1777–1793.
4. Hohmann, T.; Grabiec, U.; Ghadban, C.; Feese, K.; Dehghani, F. The influence of biomechanical properties and cannabinoids on tumor invasion. *Cell Adh. Migr.* **2016**, *11*: 54–67. doi:10.1080/19336918.2016.1183867.
